# Lightweight Multispectral Detection and DEM-Constrained Ray Consistency Localization for UAV-Assisted Search and Rescue

**DOI:** 10.3390/s26154975

**Published:** 2026-08-05

**Authors:** Yanrui Bai, Changsheng Zhu

**Affiliations:** 1 College of Intelligent Equipment, Shandong University of Science and Technology, Tai’an 271019, China; 202322090401@sdust.edu.cn; 2 Industrial Big Data Intelligent Perception and Security Technology Innovation Laboratory, Shandong University of Science and Technology, Tai’an 271019, China

**Keywords:** UAV-assisted search and rescue, multispectral object detection, RGB–infrared fusion, DEM-constrained localization, ray consistency optimization

## Abstract

Reliable target detection and geographic localization are critical for unmanned aerial vehicle (UAV)-assisted search and rescue (SAR) yet remain challenging in complex outdoor environments. Small targets in UAV Red–Green–Blue–Infrared (RGB–IR) imagery suffer from background clutter, occlusion, low illumination, and infrared thermal diffusion, while localization is vulnerable to unstable viewpoints and terrain-induced ray uncertainty. This study presents an integrated UAV-SAR framework coupling lightweight multispectral detection with Digital Elevation Model (DEM)-constrained geographic localization. For detection, the Asymmetric Fusion and Context-aware Detection (AFC-Det) network leverages asymmetric dual-stream encoding, cross-modal mutual prompting, and high-resolution anchored aggregation to enhance small-target representation from RGB–IR pairs. For localization, the Global Context-Regularized Huber Ray Consistency Optimization (GCR-HRCO) improves geolocation via global ray aggregation, multi-ray geometric consistency, Huber robust optimization, and DEM-based terrain constraints. Experimental results demonstrate AFC-Det achieves 45.4% average precision (AP) and 44.7% AP for small objects (APs) on the VTSaR dataset, with 1.7 million parameters, 8.0 GFLOPs, and 107.2 FPS, generalizing well to M3FD (54.6% AP). On SAR-DAG_raycast, GCR-HRCO reduces mean horizontal error from 6.85 m to 3.31 m and RMSE from 8.16 m to 4.33 m. Collectively, these results demonstrate the effectiveness of the proposed detection and localization components.

## 1. Introduction

Unmanned aerial vehicles (UAVs) have emerged as critical platforms for Search and Rescue (SAR) operations due to their ability to rapidly access hazardous, remote, or post-disaster environments and deliver aerial observations for emergency decision-making. Recent studies on UAV-based perception and aerial object detection indicate that SAR-oriented UAV systems are transitioning from simple image acquisition toward integrated robotic platforms that combine sensing, detection, localization, and mission-level decision support [1,2,3,4]. In such systems, two capabilities are especially critical: robust target perception and accurate geographic localization.

Deep learning has substantially advanced aerial object detection through high-resolution feature modeling, transformer-based frameworks, sparse-query mechanisms, and efficient architectures [5,6,7,8,9,10,11,12,13,14,15,16]. For instance, transformer-based detectors improve long-range feature interaction, whereas sparse-query and efficient detection frameworks reduce redundant computation for high-resolution aerial imagery. However, UAV-SAR targets are often small, weakly textured, partially occluded, and embedded in complex backgrounds. Existing studies on small-object and aerial detection highlight that high-resolution spatial details, foreground-aware computation, and scale-sensitive representations are crucial for tiny target perception [12,13,14,15,16]. Nevertheless, these designs often introduce additional computational costs, thereby limiting their applicability to resource-constrained UAV platforms.

Multispectral Red–Green–Blue–Infrared (RGB–IR) perception offers an effective solution for robust target detection under low illumination, occlusion, and visually degraded conditions. RGB images typically contain texture, boundary, color, and structural information, while infrared images provide thermal saliency that can highlight human or vehicle targets when visible-light cues are unreliable. Therefore, the key challenge in multispectral detection lies not only in fusing two modalities, but also in suppressing unreliable modality responses and preserving complementary information for small or weak targets.

Kim et al. [17] proposed uncertainty-guided cross-modal learning to mitigate the impact of unreliable visible or infrared features in pedestrian detection. This improves robustness under single-modality degradation, but it primarily focuses on ground-view pedestrian scenes and does not adequately address UAV-specific challenges such as tiny target size, large viewpoint variation, and rapidly changing backgrounds. Kim et al. [18] further introduced a memory-based cued-recall mechanism to enhance small-scale pedestrian representations by retrieving discriminative cues. While this strategy strengthens weak pedestrian features, its memory-based design adds extra complexity and is not specifically optimized for lightweight UAV deployment.

For UAV-oriented multispectral perception, Sun et al. [19] investigated drone-based RGB–infrared vehicle detection and proposed uncertainty-aware learning to address cross-modality inconsistency in aerial imagery. However, this work mainly targets vehicle detection, whereas SAR targets such as trapped persons typically have lower pixel occupancy and weaker boundary cues. Zhang et al. [20] studied drone-based Red–Green–Blue–Thermal (RGBT) tiny person detection, highlighting its inherent susceptibility to background clutter, infrared thermal diffusion, and insufficient high-resolution spatial details. Luo et al. [21] proposed an intramodal enhancement and cross-modal fusion network for UAV aerial RGB-IR small-object detection, demonstrating that both modality-specific refinement and robust fusion are essential. However, their relatively complex fusion structures may increase inference cost, making the trade-off between accuracy and lightweight deployment still critical.

Recent studies have also sought to improve RGB–IR interaction through more explicit cross-modal modeling. He et al. [22] proposed cross-modal conflict-aware learning to identify and suppress conflicting RGB–IR features, thereby reducing the negative influence of inconsistent modality responses. However, conflict modeling alone may still be insufficient for preserving fine boundaries under infrared thermal diffusion. Kim et al. [23] introduced language-driven multi-modal fusion for multispectral pedestrian detection, using semantic guidance to improve modality complementarity, but the additional guidance mechanism increases system complexity. Jang et al. [24] proposed cross-modal complementary learning with cosine-similarity-based channel resampling, improving channel-level modality selection; however, such mechanisms may still rely on stable feature alignment, which can be weakened in UAV imagery due to scale variation, motion, and viewpoint changes. Furthermore, while visible-thermal benchmarks [25] and datasets like MUST [26] underscore the value of multispectral UAV perception, these tracking-oriented studies primarily focus on temporal association rather than lightweight small-target detection for downstream geographic localization.

Reliable geographic localization is also a key requirement for UAV-assisted SAR because detected targets must be converted from image coordinates into geographic positions to support rescue decision-making. Existing studies have mainly explored UAV localization from two perspectives. One line of work uses satellite imagery, topographic information, or pose-aware benchmarks to improve UAV visual localization under Global Positioning System (GPS)-denied, oblique-view, or Six-Degree-of-Freedom (6–DoF) conditions [27,28,29]. Another line of work focuses on cross-view geo-localization, where drone, ground, and satellite images are matched through multi-view benchmarks, transformer-based correspondence learning, hard negative sampling, and geometric disentanglement [30,31,32,33,34]. These studies indicate that geographic priors and multi-view geometry are effective for improving localization robustness. However, most existing methods mainly address image-level place recognition or UAV pose estimation, while target-level geographic localization after visual detection remains underexplored. In UAV-assisted SAR scenarios, localization is further affected by detection deviations, unstable viewpoints, terrain relief, and uncertain ray–terrain intersections. Therefore, robust integration of visual detection uncertainty, global observation context, multi-ray geometric consistency, and Digital Elevation Model (DEM)-based terrain constraints remain necessary.

Motivated by these considerations, this study proposes an integrated UAV-assisted SAR framework that combines lightweight multispectral target detection with DEM-constrained geographic localization. In the visual perception stage, the Asymmetric Fusion and Context-aware Detection (AFC-Det) network is developed to robustly extract small targets from degraded RGB-IR imagery. Subsequently, the Global Context-Regularized Huber Ray Consistency Optimization (GCR-HRCO) converts these visual detections into geographic coordinates by integrating global multi-ray geometric consistency with robust Huber optimization under DEM constraints.

The main contributions of this study are summarized as follows.

1. A unified perception-to-localization framework for UAV-assisted SAR.
  We propose an integrated pipeline that links lightweight RGB-IR target detection with DEM-constrained geographic localization. Unlike studies that focus only on image-level detection or UAV pose recognition, our framework directly converts multispectral visual detections into target-level geographic coordinates through camera-ray geometry and terrain constraints.
2. Lightweight cross-modal small-target perception.
  The developed AFC-Det network robustly extracts tiny targets from degraded RGB-IR imagery. Instead of treating visible and infrared images symmetrically, AFC-Det adopts modality-specific feature encoding and early cross-modal calibration to preserve RGB structural details and infrared thermal saliency with low computational cost.
3. Geometry-consistent ray optimization for DEM-constrained localization.
  The GCR-HRCO pipeline is proposed to improve target geolocation from AFC-Det detections. By aggregating flight-level observations, fitting multiple camera rays with geometric consistency, and reducing the influence of inconsistent rays, the method improves localization stability under detection deviations, weak viewing geometry, and coarse DEM terrain constraints.

The remainder of this paper is organized as follows. Section 2 details the proposed unified framework, comprising the AFC-Det perception network and the GCR-HRCO localization pipeline. Section 3 presents the experimental settings, detection comparison results, ablation studies, generalization evaluation, and localization visualization analyses. Section 4 concludes the paper and discusses future research directions.

## 2. Materials and Methods

### 2.1. Integrated Detection and Localization Framework

**Figure 1:**
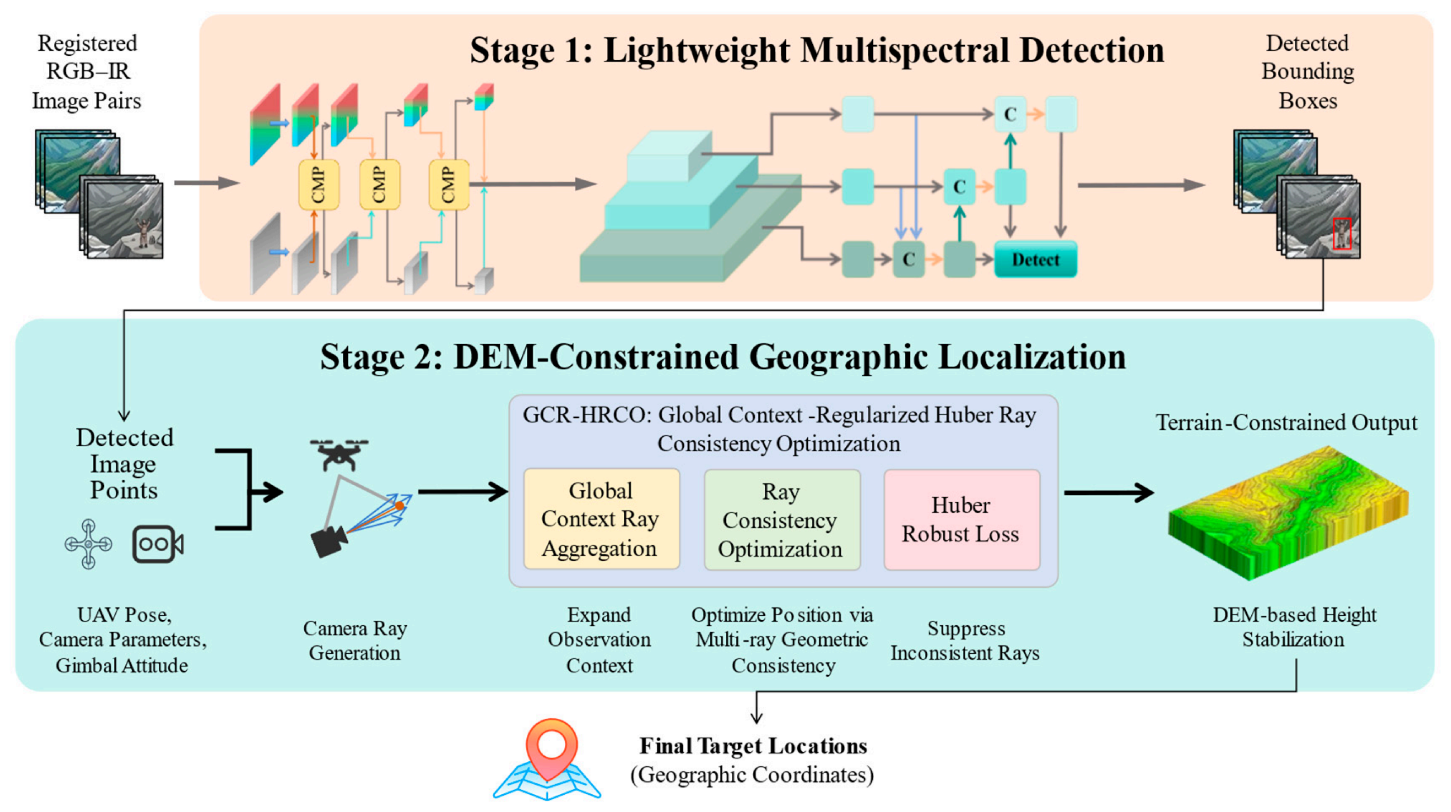
Integrated detection and localization framework.

This study presents a UAV-assisted SAR framework that couples lightweight multispectral target detection with DEM-constrained geographic localization, as shown in Figure 1. The framework adopts a perception-to-localization pipeline, where registered RGB–IR image pairs are first converted into target-level visual observations and then transformed into geographic coordinates through camera-ray geometry and terrain constraints.

In the first stage, AFC-Det detects small targets from registered RGB–IR image pairs. It adopts an asymmetric dual-stream backbone to model modality-specific RGB structural cues and infrared thermal responses, introduces the Cross-modal Mutual Prompting (CMP) module to enhance early-stage cross-modal calibration, and employs the High-Resolution Anchored Global Aggregation Pyramid (HRAG-FPN) to preserve fine spatial details for small-target representation. This stage significantly improves the reliability of visual measurements against complex environmental interferences and modality-specific degradations.

In the second stage, the detected image point is converted into a camera ray using UAV pose, camera parameters, and gimbal attitude. Rather than treating localization as an independent single-frame ray–terrain intersection problem, the proposed GCR-HRCO uses camera rays as a geometry-aware intermediate representation to link visual perception and geographic localization. Specifically, Global Context Ray Aggregation expands the observation set from a short sequence to the flight-level context, Ray Consistency Optimization estimates the target position through multi-ray geometric fitting, and Huber Robust Loss suppresses inconsistent rays during optimization. Finally, a fixed Terrain-Constrained Output step stabilizes the target height using DEM-based raycast intersections and outputs the final geographic coordinates.

### 2.2. Asymmetric Fusion and Context-Aware Detection Network

**Figure 2:**
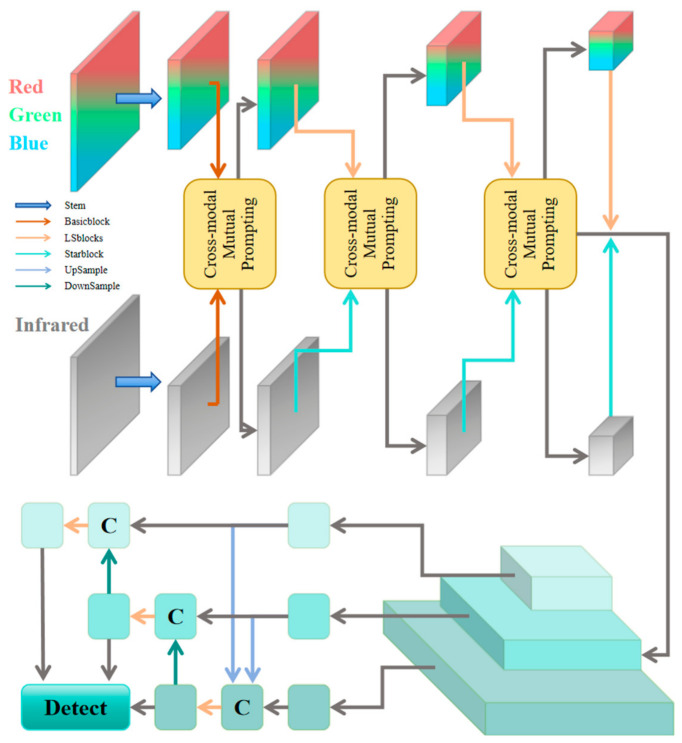
AFC-Det network structure.

In UAV-assisted search and rescue (UAV-SAR) imagery, targets typically occupy only a small image region and exhibit weak texture, limited semantic cues, and scale variation, while simultaneously suffering from background clutter, canopy occlusion, low illumination, and infrared thermal diffusion. These factors compromise both detection accuracy and the reliability of two-dimensional visual priors for subsequent geographic localization. To address these challenges, we propose AFC-Det, a lightweight multispectral detector that predicts target bounding boxes and categories from registered RGB-IR image pairs, as illustrated in Figure 2.

AFC-Det adopts an asymmetric dual-stream architecture to capture modality-specific cues. The RGB branch uses StarBlock to enhance texture, edge, and local structural representation, whereas the infrared branch employs LSBlockS to model thermal response distributions and suppress irrelevant thermal noise. Compared with symmetric dual-stream designs, this asymmetric encoding better preserves discriminative information from each modality in complex rescue environments.

To reduce spatial response shifts and semantic inconsistency caused by independent down sampling, the CMP module is introduced in shallow and intermediate stages. Specifically, the infrared-to-visible thermal saliency prompt guides RGB features using infrared spatial responses, improving target awareness under low-light or occluded conditions. Meanwhile, the visible-to-infrared structural prompt uses high-frequency RGB structural cues to constrain infrared boundary ambiguity caused by thermal diffusion. This bidirectional prompting improves cross-modal alignment while maintaining complementary modality information.

The CMP-enhanced multi-scale features are then fed into the HRAG-FPN. By aggregating high-resolution shallow details with deep semantic features and feeding fine-grained spatial cues back to high-level layers, the HRAG-FPN strengthens small-target localization and alleviates detail loss caused by repeated downsampling. Consequently, AFC-Det improves detection robustness against these severe environmental and modality-specific degradations while maintaining a lightweight structure.

#### 2.2.1. Asymmetric Dual-Stream Backbone

AFC-Det adopts an asymmetric dual-stream backbone to separately encode RGB and infrared images, enabling each modality to be processed according to its own imaging characteristics. In UAV-SAR scenarios, RGB images provide rich texture, edge, and structural details, but are easily degraded by low illumination, shadows, occlusion, and background clutter. Infrared images are more sensitive to thermal targets, but often suffer from weak boundaries, background heat interference, and thermal diffusion. Therefore, the RGB branch is designed to enhance texture and structural representation, whereas the infrared branch focuses on thermal response selection and local saliency enhancement.

In the RGB branch, StarBlock is used as the basic encoding unit. Given an input feature X, the StarBlock operation can be formulated as

(1) $$X^{\prime }=B({DWConv}_{7\times 7}(X))$$

(2) $$Z=ReLU6({Conv}_{1\times 1}f1(X^{\prime }))⊗{Conv}_{1\times 1}f2(X^{\prime })$$

(3) $$Xout=SiLU({DWConv}_{7\times 7}(B({Conv}_{1\times 1}g(Z)))+S(X))$$

where ⊗ denotes element-wise multiplication, and $S\left(X\right)$ denotes the shortcut branch. When downsampling is required, $S\left(X\right)$ consists of average pooling and a 1×1 convolution for spatial and channel alignment; otherwise, an identity shortcut is used. For the basic mathematical operators, $B\left(\cdot \right)$ denotes the Batch Normalization operation, $Conv_{1\times 1}\left(\cdot \right)$ denotes a 1×1 standard pointwise convolution, and $DWConv_{7\times 7}\left(\cdot \right)$ represents a 7×7 depthwise convolution. Specifically, $Conv_{1\times 1}f1\left(\cdot \right)$, $Conv_{1\times 1}f2\left(\cdot \right)$, and $Conv_{1\times 1}g\left(\cdot \right)$ denote three distinct 1×1 convolutional layers with independent learnable weights used for channel expansion, interaction, and projection, respectively. Furthermore, $\mathrm{ReLU}6\left(\cdot \right)$ represents the ReLU6 activation function, and $SiLU\left(\cdot \right)$ denotes the SiLU activation function.

**Figure 3:**
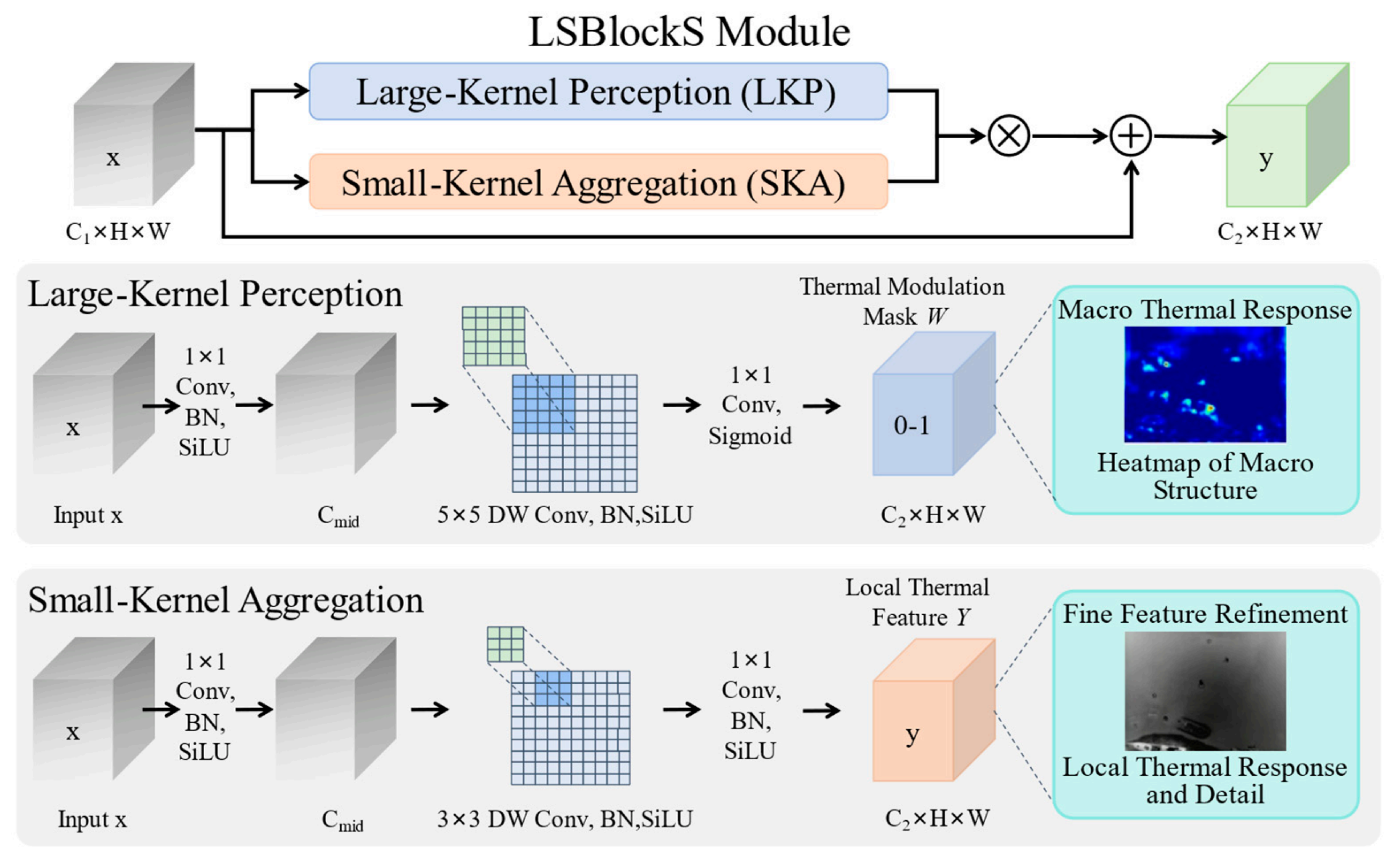
Illustration of the LSBlockS module.

We introduce LSBlockS into the infrared branch to model locally concentrated thermal responses while suppressing background thermal noise, and its structure is shown in Figure 3. Given the input feature X, LSBlockS contains a Large-kernel Perception (LKP) branch and a Small-kernel Aggregation (SKA) branch. The LKP branch generates a thermal modulation mask, while the SKA branch aggregates local thermal details for infrared small-target representation, which are defined as

(4) $$W=\sigma \left({Conv}_{1\times 1}\left(SiLU\left(B\left({DWConv}_{5\times 5}\left(SiLU\left(B\left({Conv}_{1\times 1}\left(X\right)\right)\right)\right)\right)\right)\right)\right)$$

(5) $$Y=SiLU\left(B\left({Conv}_{1\times 1}\left(SiLU\left(B\left({DWConv}_{3\times 3}\left(SiLU\left(B\left({Conv}_{1\times 1}\left(X\right)\right)\right)\right)\right)\right)\right)\right)\right)$$

(6) $$X_{out}=X+Y⊗W$$

where W denotes the thermal modulation weight, Y denotes the local thermal feature, and $\sigma \left(\cdot \right)$ represents the Sigmoid activation function. The expansion ratio of LSBlockS is set to e=1.0, which avoids premature channel compression of infrared small-target features while maintaining a lightweight structure.

Furthermore, the selection of distinct activation functions within the StarBlock is explicitly tailored to their specific operational roles. As formulated in Equation (2), ReLU6 is employed for internal feature interaction because its bounded output range of [0,6] effectively prevents numerical explosion during the element-wise multiplication of high-dimensional features. Additionally, this bounded property provides substantial robustness for low-precision hardware quantization, such as INT8, which is beneficial for future implementation on UAV edge devices. Conversely, SiLU is utilized as the final output activation of the block, as indicated in Equation (3). Benefiting from its smooth and non-monotonic mathematical properties, SiLU alleviates the dying ReLU phenomenon, thereby ensuring stable gradient propagation and preserving fine-grained RGB structural details for subsequent cross-modal fusion.

#### 2.2.2. Cross-Modal Mutual Prompting

**Figure 4:**
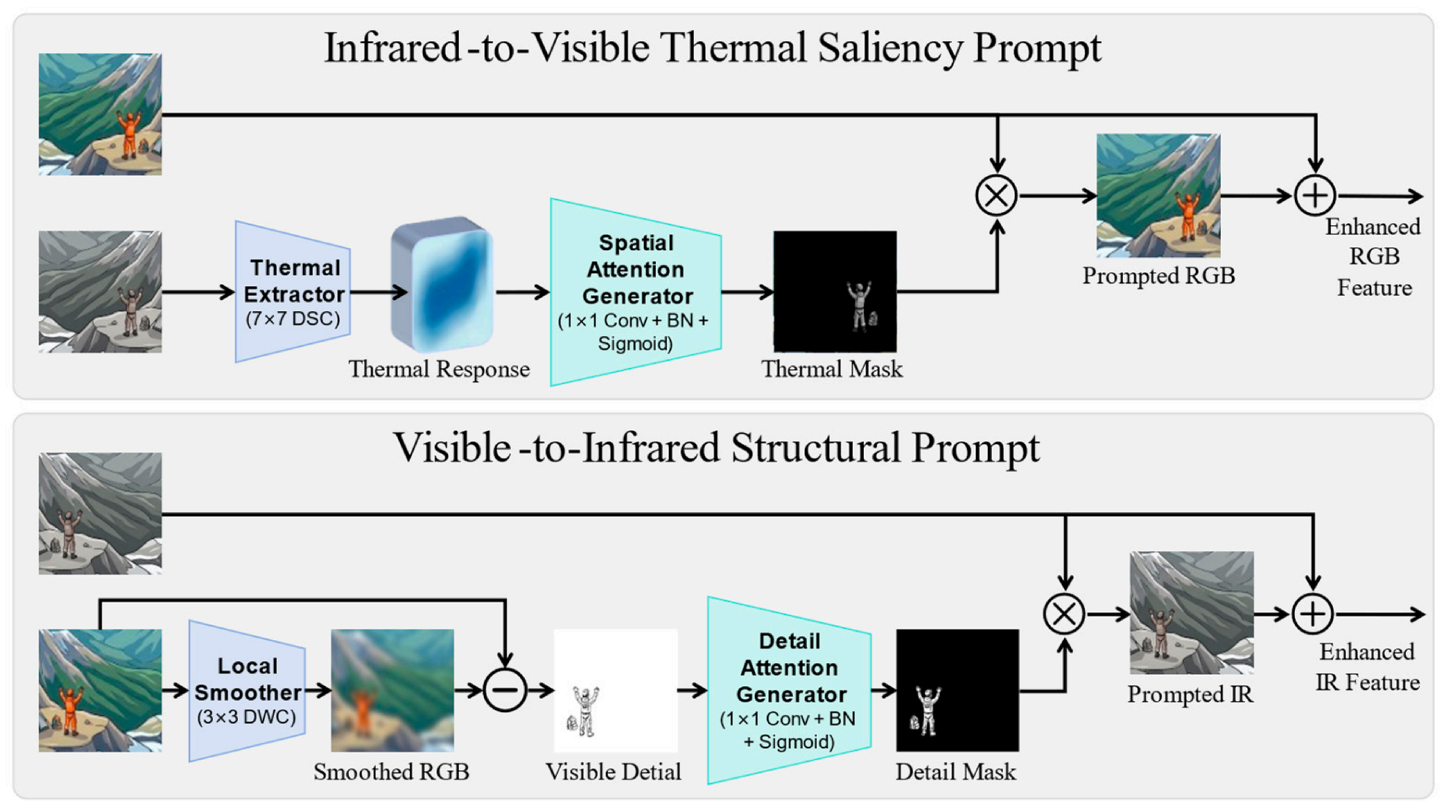
Illustration of the CMP module.

In RGB-IR dual-stream detection networks, RGB and infrared features are usually encoded separately, which may cause modality-specific noise, spatial response shifts, and semantic inconsistency during repeated downsampling. Infrared features are vulnerable to thermal diffusion and background heat interference, while RGB features can be degraded by shadows, low illumination, and complex texture clutter. Therefore, simple late-stage fusion is insufficient to correct the misalignment accumulated in intermediate encoding. To address this problem, CMP is introduced into the shallow and intermediate stages of the dual-stream backbone, enabling bidirectional modulation before the features enter the next encoding stage. The structure of CMP is illustrated in Figure 4.

Given the infrared feature $F_{ir}$ and visible feature $F_{vis}$ at the current stage, where $F_{ir},F_{vis}\in R^{C\times H\times W}$, CMP generates an infrared-to-visible thermal saliency prompt and a visible-to-infrared structural prompt as follows:

(7) $$M_{ir\to vis}=\sigma \left(B\left({Conv}_{1\times 1}\left({DSConv}_{7\times 7}\left(F_{ir}\right)\right)\right)\right)$$

(8) $$D_{vis}=F_{vis}-{DWConv}_{3\times 3}\left(F_{vis}\right)$$

(9) $$M_{vis\to ir}=\sigma \left(B\left({Conv}_{1\times 1}\left(D_{vis}\right)\right)\right)$$

where $M_{ir\to vis}$ provides thermal saliency guidance for the RGB branch, and $M_{vis\to ir}$ provides structural constraints for the infrared branch. For the operators and intermediate variables, $\sigma \left(\cdot \right)$ represents the standard sigmoid activation function, and $DSConv_{7\times 7}\left(\cdot \right)$ denotes a 7×7 depthwise separable convolution layer. Additionally, the term $D_{vis}$ explicitly represents the extracted high-frequency structural detail feature from the visible modality. The bidirectional prompting process is then formulated as

(10) $$F_{vis}^{\prime }=F_{vis}⊗\left(1+M_{ir\to vis}\right)$$

(11) $$F_{ir}^{\prime }=F_{ir}⊗\left(1+M_{vis\to ir}\right)$$

By introducing CMP before subsequent encoding stages, cross-modal interaction is performed earlier, effectively reducing modality misalignment and noise accumulation during feature extraction.

#### 2.2.3. High-Resolution Anchored Global Aggregation Pyramid

**Figure 5:**
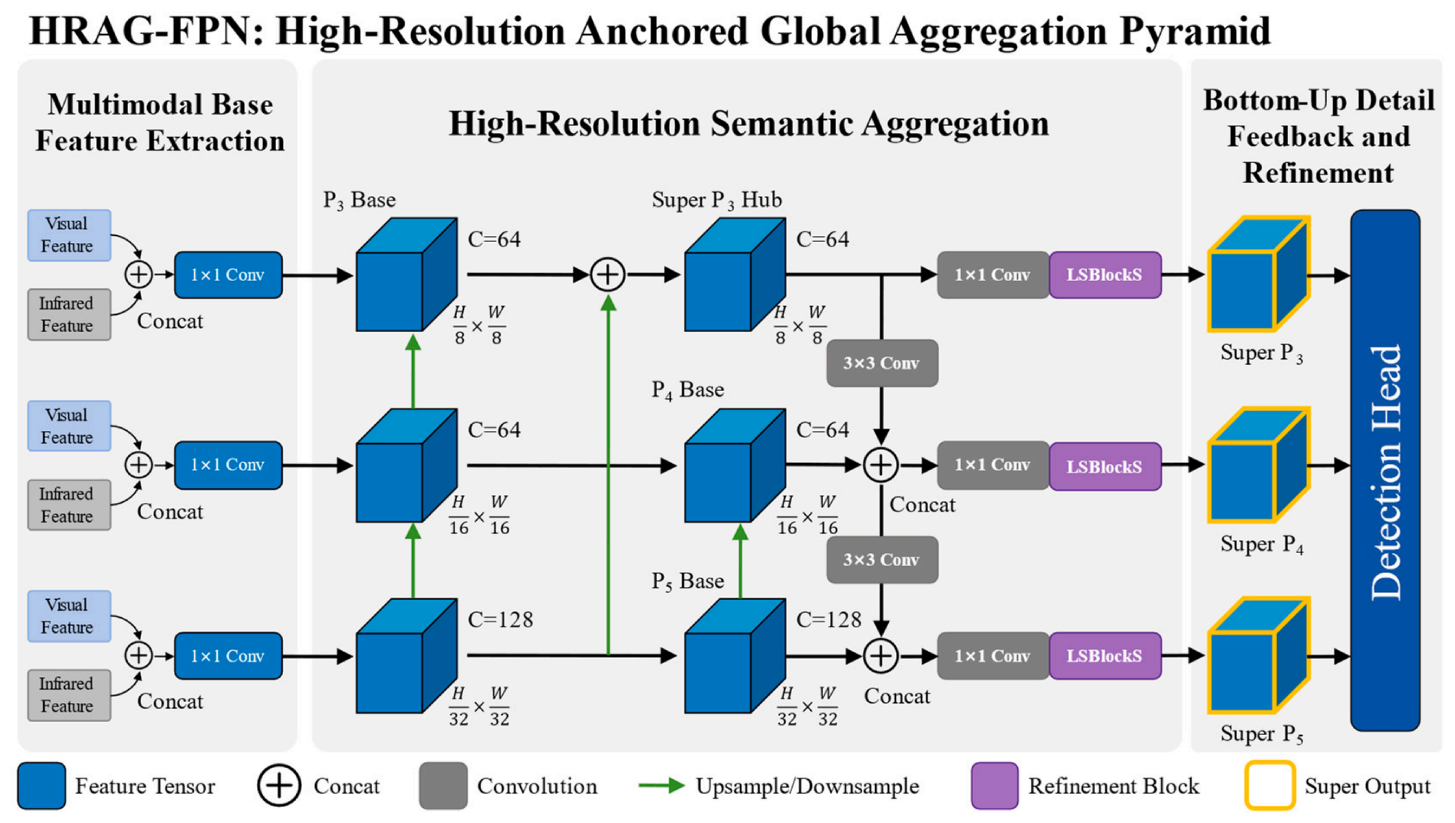
Illustration of the HRAG-FPN module.

In UAV-SAR scenarios, small targets often suffer from background interference and low pixel occupancy. Conventional Feature Pyramid Networks (FPNs) progressively downsample feature maps and may lose crucial shallow spatial details, such as object boundaries, local textures, and fine target cues. To address this issue, we propose the HRAG-FPN, whose structure is illustrated in Figure 5.

HRAG-FPN uses the high-resolution $P3_{\mathrm{base}}$ feature as a global aggregation hub to enhance small-target representation. Specifically, multi-scale base features $P3_{\mathrm{base}}$, $P4_{\mathrm{base}}$, and $P5_{\mathrm{base}}$ are first extracted from the multi-modal backbone features. Deep semantic information from $P4_{\mathrm{base}}$ and $P5_{\mathrm{base}}$ is then directly aggregated into $P3_{\mathrm{base}}$, forming an enhanced high-resolution hub representation. This hub-based aggregation shortens the cross-scale information transmission path, reduces semantic attenuation, and preserves fine spatial details for small-target feature localization.

After high-resolution semantic aggregation, the enhanced $P_{3}$ representation is propagated back to deeper feature levels through a bottom-up residual feedback pathway. This feedback mechanism compensates for spatial detail loss in low-resolution features while preserving their semantic discrimination. Each enhanced pyramid feature is further refined by a lightweight LSBlockS-based refinement block before being fed into the detection head. In this way, HRAG-FPN provides stable and discriminative multi-scale features for AFC-Det, thereby improving small-target detection and bounding-box localization under complex UAV-SAR backgrounds.

### 2.3. Vision-Guided DEM-Constrained Ray Consistency Localization

In UAV-SAR scenarios, target localization from image detections requires converting image-space observations into geographic coordinates. A conventional raycast-based pipeline first converts the detected image point into a camera ray and then intersects the ray with a DEM terrain surface. In practical SAR missions, especially in remote mountainous or disaster-affected regions, publicly available DEMs provide an accessible and efficient terrain in advance without requiring additional field surveying or high-cost terrain reconstruction. Therefore, DEM-constrained localization offers a practical solution for rapidly estimating target positions in complex outdoor environments.

**Figure 6:**
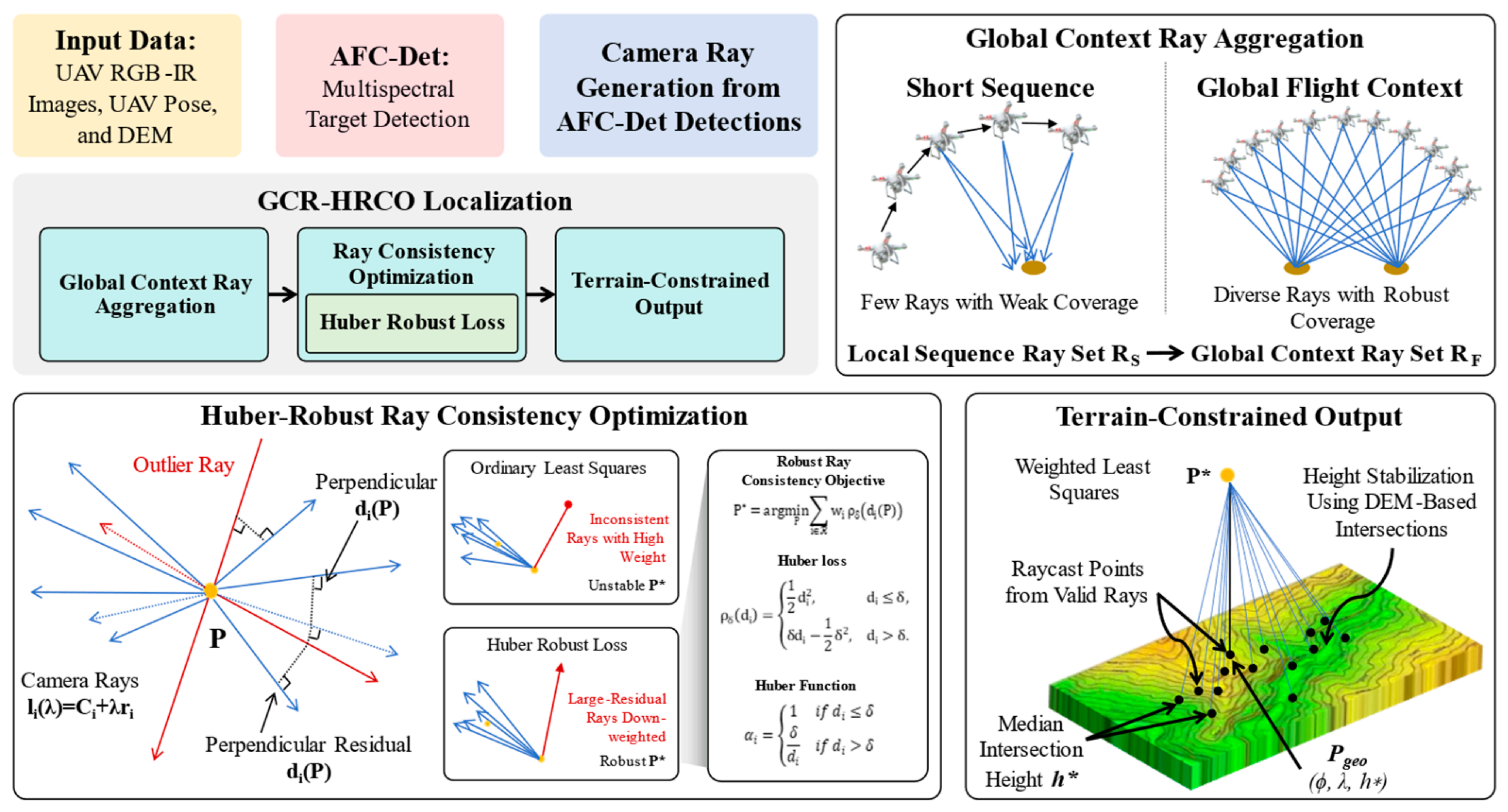
Illustration of the overall architecture of the proposed localization framework.

However, localization based on independent single-frame ray–terrain intersections can still be sensitive to bounding-box deviations, viewpoint changes, and local terrain discretization. When localization is performed independently for each frame or simply averaged within a short sequence, the final position may be affected by weak viewing geometry and inconsistent ray observations. To address this issue, this study formulates target geolocation as a DEM-constrained multi-ray consistency problem. Instead of relying on a single raycast intersection, the proposed method aggregates multiple camera rays and estimates a geometrically consistent target position under terrain constraints. The overall architecture of the proposed localization framework is illustrated in Figure 6.

To improve the stability of DEM-constrained target localization, we propose the GCR-HRCO method. The method comprises three independent modules: Global Context Ray Aggregation, Ray Consistency Optimization, and Huber Robust Loss. The Global Context Ray Aggregation module expands the observation set from a short sequence to the flight-level context, thereby providing richer multi-view geometric constraints. The Ray Consistency Optimization module determines the target position by fitting multiple camera rays instead of averaging independent DEM intersections. The Huber Robust Loss function further reduces the influence of inconsistent rays during optimization. Finally, a Terrain-Constrained Output step uses DEM-based raycast intersections to stabilize the vertical coordinate and yield the final geographic position.

#### 2.3.1. Camera Ray Generation from AFC-Det Detections

For each UAV frame, the target bounding box is directly obtained from AFC-Det predictions. Let the predicted bounding box in the i-th frame be

(12) $$B_{i}^{det}=(x_{i1}^{det},y_{i1}^{det},x_{i2}^{det},y_{i2}^{det})$$

where $\left(x_{i1}^{det},y_{i1}^{det}\right)$ and $\left(x_{i2}^{det},y_{i2}^{det}\right)$ denote the upper-left and lower-right coordinates. The image point for ray generation is defined as the bottom-center point of the bounding box to approximate the target ground-contact position:

(13) $$u_{i}=\frac{x_{i1}^{det}+x_{i2}^{det}}{2}$$

(14) $$v_{i}=y_{i2}^{det}$$

Given the camera intrinsic parameters $\left(f_{x},f_{y},c_{x},c_{y}\right)$, the image point is converted into normalized camera coordinates:

(15) $$x_{i}=\frac{u_{i}-c_{x}}{f_{x}}$$

(16) $$y_{i}=\frac{v_{i}-c_{y}}{f_{y}}$$

Therefore, the corresponding unit ray direction vector $r_{i}$ within the local camera coordinate system is formulated as

(17) $${\overset{\sim }{r}}_{i}=\frac{{\left[x_{i},y_{i},1\right]}^{T}}{\left|[x_{i},y_{i},1{]}^{T}\right|}$$

Using the UAV position, flight attitude, camera parameters, and gimbal metadata, the camera-frame ray is transformed into the world coordinate system. This produces a camera center $C_{i}\in R^{3}$ and a unit ray direction $r_{i}\in R^{3}$. The camera ray is written as

(18) $$l_{i}(t)=C_{i}+tr_{i},t>0$$

where t denotes the parametric scalar representing the distance along the camera ray.

#### 2.3.2. Global Context Aggregation and Multi-Ray Consistency

A short sequence may contain only a limited number of valid observations, leading to unstable localization when the viewing geometry is weak. Therefore, instead of using only the current sequence, GCR-HRCO aggregates rays from a broader flight-level context. For a given sequence s belonging to a flight F, let the local sequence ray set be

(19) $$R_{s}=\left(C_{i},r_{i}\right),i\in s$$

Moreover, the global context ray set is defined as

(20) $$R_{F}=\left(C_{i},r_{i}\right),i\in F$$

Since each flight corresponds to the same static target in the evaluated SAR-DAG setting, $R_{F}$ provides richer multi-view geometric constraints than $R_{s}$. The Global Context Ray Aggregation module replaces the local ray set with the flight-level ray set when estimating the final target position.

#### 2.3.3. Ray Consistency Optimization

Ray Consistency Optimization estimates a three-dimensional target hypothesis P by minimizing its perpendicular distances to multiple observation rays. For the i-th ray, the perpendicular residual is defined as

(21) $$d_{i}\left(P\right)={∥\left(I-r_{i}r_{i}^{T}\right)\left(P-C_{i}\right)∥}_{2}\;$$

where $I\in R^{3\times 3}$ represents the identity matrix, and $\left(I-r_{i}r_{i}^{T}\right)$ projects the vector $\left(P-C_{i}\right)$ onto the plane perpendicular to the ray direction $r_{i}$. Therefore, $d_{i}\left(P\right)$ measures the shortest distance from the candidate target point to the camera ray.

Without the Huber robust loss, the ray consistency objective is formulated as a weighted least-squares problem:

(22) $$P^{*}=arg\;\underset{P}{min}[\frac{1}{2}\sum_{i\in R}w_{i}d_{i}^{2}(P)+\frac{\gamma }{2}{∥P∥}_{2}^{2}]$$

where $P\in R^{3}$ denotes the estimated three-dimensional target position, and ‖⋅‖ _2_ signifies the standard $L_{2}$ norm. R denotes either the sequence-level ray set $R_{s}$ or the flight-level ray set $R_{F}$, depending on whether Global Context Ray Aggregation is enabled. The weight $w_{i}$ reflects the observation quality associated with the bounding box, and γ represents the Tikhonov regularization coefficient used to improve numerical stability.

#### 2.3.4. Huber Robust Loss and Terrain-Constrained Output

Although Ray Consistency Optimization improves localization by jointly fitting multiple rays, ordinary least-squares fitting can still be affected by inconsistent rays. Therefore, the Huber Robust Loss is introduced to reduce the influence of large-residual observations. For the residual $d_{i}\left(P\right)$, the Huber loss is defined as

(23) $$\rho_{\delta }\left(d_{i}\right)=\left\{\begin{array}{cc} \frac{1}{2}d_{i}^{2}, & d_{i}\leq \delta \\ \delta d_{i}-\frac{1}{2}\delta^{2}, & d_{i}>\delta \end{array}\right.$$

The robust ray consistency objective is then written as

(24) $$P^{*}=arg\;\underset{P}{min}[\sum_{i\in R}w_{i}\rho_{\delta }\left(d_{i}\left(P\right)\right)+\frac{\gamma }{2}{∥P∥}_{2}^{2}]$$

where $\rho_{\delta }\left(\cdot \right)$ represents the Huber robust loss function, and δ denotes the threshold hyperparameter that differentiates inlier observations from outlier ray residuals.

To ensure full reproducibility, the inherently non-linear Huber-based objective function in Equation (24) is optimized using the Iteratively Reweighted Least Squares (IRLS) algorithm. First, the IRLS optimization is triggered only when a minimum of 3 valid camera rays are successfully generated; otherwise, the system robustly defaults to the spatial median of the independent DEM raycast intersections. To prevent divergence caused by severe outliers or near-parallel rays during UAV hovering, the optimization is initialized with a robust warm start, $P_{0}$, defined as the spatial median of the valid DEM intersections. Furthermore, a hard spatial boundary constraint is enforced during iteration: if the horizontal position update shifts by more than 25.0 m from the initial median, the step is dynamically clipped.

Second, the objective function in Equation (24) employs a dynamic-static composite weighting strategy at step k, where the overall weight $w_{i}^{\left(k\right)}$ is formulated as the product of a static observation quality weight $q_{i}$ and a dynamic robust factor $\alpha_{i}^{\left(k\right)}$. The dynamic factor $\alpha_{i}^{\left(k\right)}$ is computed at the k-th iteration based on the perpendicular residual distance $d_{i}^{\left(k-1\right)}$ between the i-th camera ray and the previous spatial estimate $P^{\left(k-1\right)}$, defined as follows:

(25) $$\alpha_{i}^{\left(k\right)}=\left\{\begin{array}{cc} 1, & \mathrm{if}\;d_{i}^{\left(k-1\right)}\leq \delta \\ \frac{\delta }{d_{i}^{\left(k-1\right)}}, & \mathrm{if}\;d_{i}^{\left(k-1\right)}>\delta \end{array}\right.$$

where δ=3.0 m denotes the empirical Huber robust threshold aligning with nominal civilian UAV GNSS errors. When $d_{i}^{\left(k-1\right)}>\delta$, $\alpha_{i}^{\left(k\right)}$ smoothly transitions the loss from quadratic $L_{2}$ to linear $L_{1}$, dynamically suppressing the influence of large-residual outlier rays.

Concurrently, the static observation quality weight $q_{i}$ remains invariant across iterations and models heteroscedastic frame-level measurement noise according to:

(26) $$q_{i}=\frac{\left|r_{i,z}\right|}{d_{i}\cdot \left(1+\epsilon_{platform}+\epsilon_{visual}\right)}$$

where $d_{i}$ represents the camera-to-target slant distance, and $\left|r_{i,z}\right|$ is the absolute vertical component of the unit ray direction, effectively reducing the weight of shallow incident angles. The terms $\epsilon_{platform}$ and $\epsilon_{visual}$ denote the aggregated angular uncertainties (in radians) derived from the UAV platform attitude fluctuations (roll, pitch, yaw) and the bounding box center perturbations, respectively. This rigorous formulation heavily penalizes distant, severely tilted, or highly vibrating visual observations, thereby preventing the geometric ‘lever-arm’ amplification of projection errors.

Finally, the optimization iterates until the spatial update is less than $10^{-4}$ m or reaches a maximum of 8 iterations. To counteract the weak vertical geometric constraint typical of aerial downward views, the *Z*-axis coordinate of the optimized 3D point is explicitly decoupled. After obtaining the optimized horizontal position, the target height $h^{*}$ is fixed to the median elevation of the valid DEM-based raycast intersections. Since the DEM used in practical UAV-SAR localization may have limited spatial accuracy, this height constraint is applied only as a fixed terrain-consistent output step rather than as an independent optimization module. This design reduces the dependence on any single ray–terrain intersection while preventing the optimized point from drifting unrealistically above or below the terrain surface. The final geographic target position is expressed as

(27) $$P_{geo}=\left(\phi ,\lambda ,h^{*}\right)$$

where ϕ and λ denote the predicted latitude and longitude coordinates, respectively, and $h^{*}$ represents the previously defined median terrain elevation.

## 3. Experimental Results and Discussion

### 3.1. Datasets and Implementation Details

In complex disaster environments, a single visible-light sensor often fails perceptually due to abrupt illumination changes. To evaluate the robustness of the proposed multi-modal fusion system, this study adopts the VTSaR [35] dataset as the experimental benchmark. This high-altitude reconnaissance dataset provides strict spatiotemporal alignment between visible-light and infrared modalities based on a dual-sensor gimbal, and its hardware-level registration ensures multi-sensor spatial errors are eliminated, offering reliable data support for cross-modal feature fusion. In addition, VTSaR covers complex outdoor environments with varying conditions such as vegetation occlusion and low illumination, with annotations specifically focusing on trapped individuals, thereby objectively reflecting the physical constraints of real SAR operations. Validation on this dataset therefore directly reflects the model’s environmental adaptability for delivering reliable perception under low-visibility emergency scenarios.

The Multi-scenario Multi-Modality Benchmark (M3FD) [36] is utilized to evaluate multi-modal perception systems under complex weather conditions. The dataset contains 4200 pairs of pixel-level aligned RGB–IR images, covering various scenarios such as daytime, overcast conditions, nighttime, and real fog, and annotates six categories, including pedestrians and vehicles. Benefiting from this rigorous alignment, the feature-complementarity effectiveness of dual-light fusion algorithms under varying illumination and thermal radiation conditions can be systematically quantified, thereby providing an objective evaluation basis for the perceptual robustness of unmanned rescue or patrol platforms under adverse weather conditions.

The SAR-DAG_raycast dataset [37] was used to evaluate target-level geographic localization. The dataset provides UAV flight records, target bounding-box annotations, ground-truth geographic coordinates, GPS measurements, flight attitude, gimbal metadata, camera information, and DEM terrain data. After excluding combined CSV files that duplicated individually recorded flights, the evaluation subset comprised seven flights, 16 temporally continuous sequences, and 154 evaluated frames. A new sequence was initiated when the temporal gap between two consecutive valid frames exceeded 3.5 s. RGB images paired with the corresponding DR-AVIT-derived infrared representations were utilized for AFC-Det inference.

### 3.2. Evaluation of Multispectral Target Detection

The experiments were conducted on a Linux Ubuntu 22.04 operating system with an NVIDIA RTX 3090 GPU (NVIDIA Corporation, Santa Clara, CA, USA) equipped with 24 GB of memory. The programming language used was Python 3.10, and the deep learning framework was PyTorch 2.1.2 with CUDA 11.8. The main training parameters are listed in Table 1.

The evaluation subset encompassed seven flights targeting six distinct ground-truth locations. During testing, all 154 evaluated frames successfully generated valid DEM-intersecting rays, achieving a 100.0% valid-ray rate. For metric ray generation and multi-ray optimization, geographic latitude and longitude measurements were converted into a local East-North-Up coordinate frame. Detailed statistics regarding flight geometry, target distances, and specific DEM configurations are summarized in Table 2.

#### 3.2.1. Comparison with State-of-the-Art Methods

**Figure 7:**
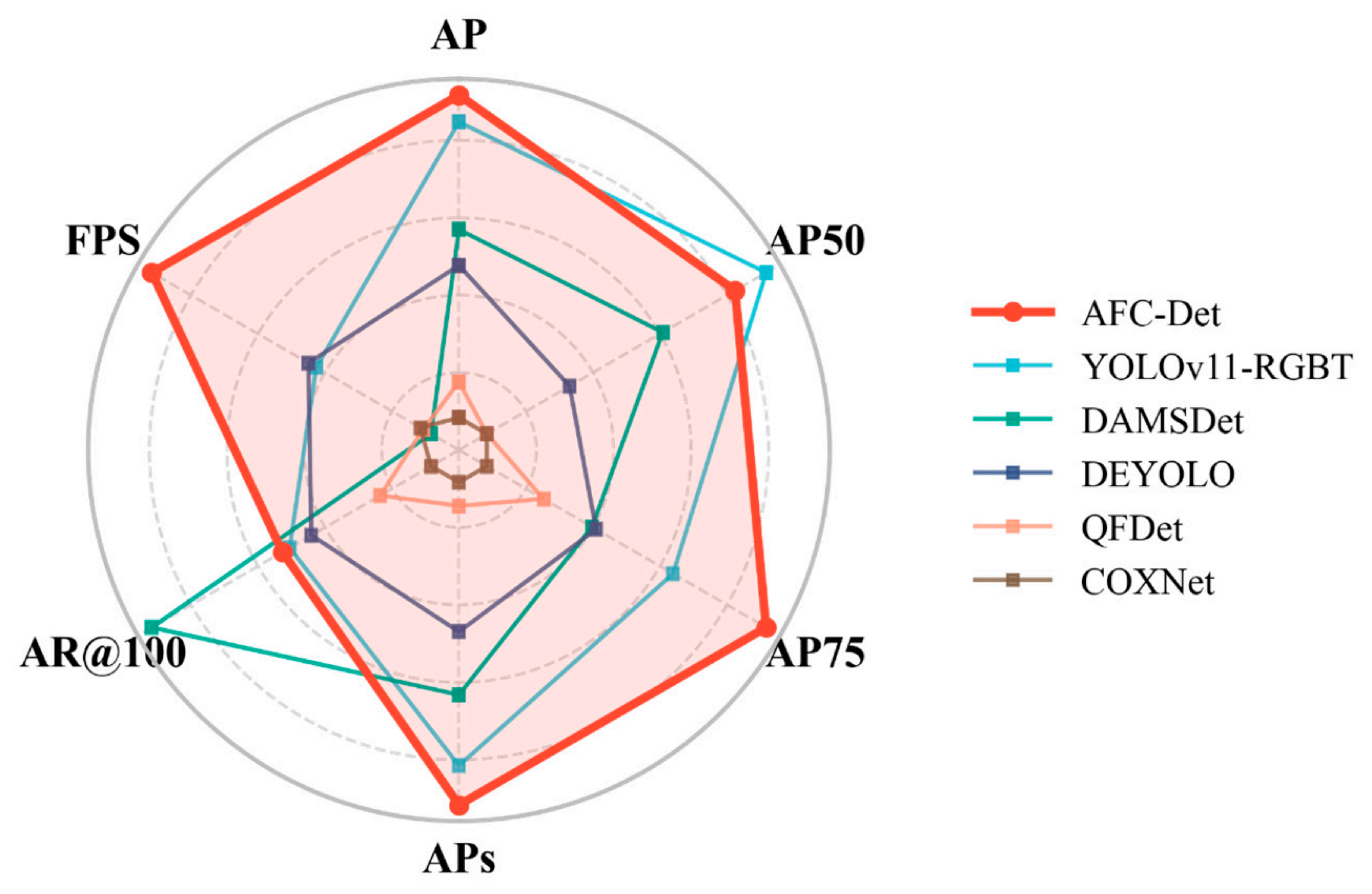
Performance comparison with state-of-the-art methods on the VTSaR dataset.

To comprehensively evaluate the detection performance of AFC-Det in complex SAR scenarios, this study compares it with several multi-modal detection methods, including YOLOv11-RGBT, DAMSDet, DEYOLO, QFDet, and COXNet, on the VTSaR dataset. As shown in Table 3 and Figure 7, AFC-Det achieves superior performance in detection accuracy, small-object perception capability, and lightweight architectural efficiency. To ensure a fair evaluation, the detection models were retrained on the VTSaR dataset using their official implementations. Instead of relying on pre-published weights, we initialized all models with COCO pre-trained weights and standardized the training environment. Every model shared a 640 × 640 input resolution, a 300–epoch schedule, and an effective batch size of 32, utilizing gradient accumulation to satisfy GPU memory constraints.

We applied an identical data augmentation pipeline across all configurations. This included a 1.0 probability for Mosaic, 0.1 for MixUp, 0.5 for random horizontal flip, and HSV color jittering with hue, saturation, and value thresholds of 0.015, 0.7, and 0.4, respectively. To ensure optimal convergence, we retained each model’s default optimization recipe alongside all other unspecified hyperparameters strictly according to their original official configurations. All experiments were conducted on a single NVIDIA GeForce RTX 3090 GPU.

To address concerns regarding the relatively incremental performance margins over recent baselines and to rigorously evaluate the statistical consistency of our framework, we conducted multiple independent training and evaluation trials across five distinct random seeds, namely seeds 42, 43, 44, 45, and 46. The quantitative results are reported in a unified deviation format. In this notation, the primary value represents the benchmark performance under seed 42, with upper and lower offsets indicating the maximum and minimum deviations across all five trials. This multi-run evaluation transparently illustrates the operational bounds of each network and confirms that the advantages of AFC-Det remain highly consistent and reproducible against stochastic optimization noise.

In terms of overall detection performance, AFC-Det attains an AP of 45.4%, achieving comparable accuracy to the highly competitive YOLOv11-RGBT baseline at 45.1%, as this marginal difference lies within the observed seed-to-seed variation. However, AFC-Det demonstrates a clear and statistically significant advantage under stricter evaluation criteria. Specifically, under the high IoU threshold, AFC-Det achieves an AP75 of 33.7%, outperforming YOLOv11-RGBT by a substantial margin of 2.3 percentage points, demonstrating superior bounding-box localization capability. This improvement is primarily attributed to the cross-modal feature interaction and multi-scale feature enhancement mechanisms, which enable the model to more effectively suppress redundant noise and enhance target region representation quality under complex backgrounds.

Furthermore, while the overall mean AP remains comparable as previously discussed, the deviation bounds in Table 3 validate the statistical consistency and superior stability of AFC-Det across multiple independent trials. While YOLOv11-RGBT achieves a competitive baseline AP of 45.1%, it suffers a maximum AP drop of 0.9% across different random seeds. In contrast, AFC-Det demonstrates robust lower-bound stability, restricting its maximum AP drop to merely 0.2% alongside an upward potential of 0.8%. Moreover, its performance degradations under stricter metrics such as AP75 and APs are tightly constrained to 0.2% and 0.1% respectively. This exceptional robustness indicates that AFC-Det is highly insensitive to random initialization and optimization noise, ensuring consistently reliable perception for practical UAV deployment.

Regarding model complexity, AFC-Det demonstrates an unmatched accuracy-efficiency trade-off. It contains only 1.7M parameters and 8.0 GFLOPs, while achieving a real-time inference speed of 107.2 FPS. In contrast, DAMSDet has 79.1M parameters and 134.7 GFLOPs, with an inference speed of only 21.4 FPS, and YOLOv11-RGBT requires nearly three times the parameters, specifically 5.0M, while running at half the speed at 56.7 FPS. Therefore, AFC-Det significantly reduces model size and computational overhead while delivering clear advantages in strict localization and processing speed, demonstrating a computationally lightweight architecture that paves the way for future edge deployment on resource-constrained UAV platforms.

#### 3.2.2. Ablation Studies and Component Analysis

To clarify the progression of our ablation studies and ensure reproducibility, we define a reference architecture. In Table 4, the Baseline represents a standard asymmetric dual-stream network. Structurally, it employs standard BasicBlock for feature extraction in both branches—where each block is a residual unit comprising two 3×3 convolutions and an average-pooling shortcut for downsampling. Furthermore, it fuses the multi-modal features via a simple concatenation, aggregates them utilizing a standard Feature Pyramid Network (FPN) rather than our proposed HRAG-FPN, and entirely excludes the CMP module. This baseline serves to validate the incremental contributions of our proposed components.

**Figure 8:**
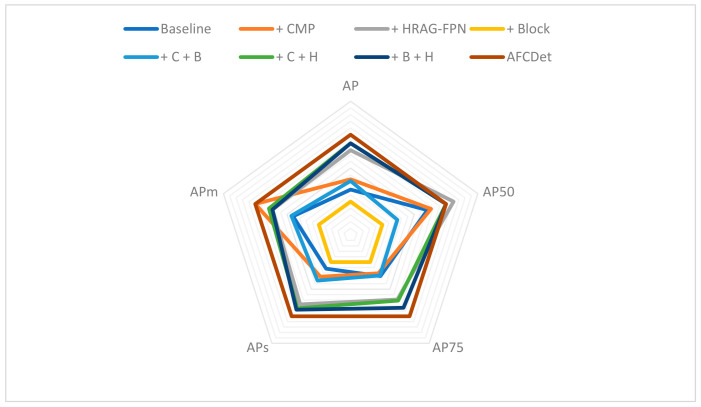
Ablation study of AFC-Det on the VTSaR dataset.

To verify the independent contributions and synergistic effects of each core module, module ablation experiments were conducted under a unified experimental setting, and the results are shown in Table 4 and Figure 8. The Baseline achieved AP, AP75, and APs values of 42.2, 26.5, and 41.1, respectively, with 2.89 M parameters and 9.4 GFLOPs.

First, introducing CMP alone marginally increased parameters to 2.95M and GFLOPs to 9.9, while boosting AP, APs, and APm to 42.8, 41.7, and 51.6, respectively. These results demonstrate that CMP can enhance cross-modal feature interaction at a small computational cost, particularly improving the semantic representation of medium-scale objects. Alternatively, solely applying HRAG-FPN raised AP to 44.5, alongside significant improvements in AP75 and APs, showing that the high-resolution anchored multi-scale aggregation structure can effectively improve small-object feature representation and boundary localization capability. In contrast, replacing only the feature encoding block with our lightweight design substantially reduced parameters to 1.35M and GFLOPs to 7.1, though AP slightly dropped to 41.5. This suggests that the lightweight encoding block can substantially reduce model complexity, yet still requires the assistance of cross-modal calibration and multi-scale aggregation modules when used alone.

The dual-module combination experiments further demonstrate clear complementarity among the modules. By combining CMP with the lightweight encoding block, the model achieved an AP of 42.7 with 1.41M parameters and 7.6 GFLOPs, showing a clear recovery over using the lightweight block alone. This indicates that cross-modal calibration can provide more stable inputs for lightweight feature extraction. Coupling CMP and HRAG-FPN yielded 44.9 AP, but at a relatively high computational overhead of 3.55M parameters and 11.4 GFLOPs. Conversely, pairing the lightweight encoding block with HRAG-FPN matched the 44.9 AP while maintaining an efficient 1.59M parameters and 7.5 GFLOPs, alongside superior AP75 and APs scores. This indicates good compatibility between lightweight feature extraction and high-resolution multi-scale aggregation.

Finally, the complete AFC-Det integrates CMP, HRAG-FPN, and the lightweight modality-specific encoding block, achieving the best overall performance. Compared with the Baseline, AFC-Det improves AP, AP75, and APs to 45.4, 33.7, and 44.7, corresponding to relative gains of 7.6 percent, 27.2 percent, and 8.8 percent, respectively. Meanwhile, it requires only 1.65M parameters and 8.0 GFLOPs, successfully surpassing the baseline efficiency. These results confirm that AFC-Det effectively reduces model complexity while improving detection accuracy and small-object localization capability, validating the effectiveness of the synergistic design of cross-modal calibration, multi-scale aggregation, and lightweight feature encoding.

In Table 5, the goal is specifically to isolate and evaluate the effectiveness of our modality-specific lightweight feature extractors (StarBlock and LSBlockS). Therefore, the BasicBlock Variant in Table 5 is actually the complete architecture including both the CMP and HRAG-FPN modules, but utilizing the aforementioned standard BasicBlocks instead of our proposed lightweight blocks. This setting corresponds exactly to the + C + H variant in Table 4.

As shown in Group A of Table 5, when both branches adopt BasicBlock, the model achieves an AP of 44.9 and an AP75 of 30.9. However, this conventional convolutional stacking exhibits relatively evident computational redundancy, requiring 3.551M parameters and 11.4 GFLOPs.

Introducing StarBlock solely into the RGB branch marginally reduces parameters to 2.579M and GFLOPs to 9.1 while improving AP75 to 31.3 and APm to 52.0. This indicates enhanced structural and texture representation for the visible-light branch at a lower computational overhead. Conversely, applying LSBlockS exclusively to the Thermal branch further reduces parameters to 2.263M but degrades AP, AP75, and APs to 44.2, 28.9, and 43.3 respectively. This performance drop occurs because infrared images lack clear boundaries, meaning the thermal-response selection of LSBlockS strictly requires complementary structural priors from the RGB branch to achieve stable gains.

Applying StarBlock and LSBlockS simultaneously yields the best overall performance. Compared with the BasicBlock Variant, this standard configuration slashes parameters by 53.5 percent and GFLOPs by 29.8 percent, while boosting AP, AP75, and APs to 45.4, 33.7, and 44.7 respectively. These results confirm their strong cross-modal complementarity. StarBlock enhances spatial-structure representation in the RGB branch, whereas LSBlockS suppresses background thermal noise in the Thermal branch, collectively improving high-IoU localization and small-object detection with significantly reduced computational cost.

Furthermore, Group B compares different structural variants of LSBlockS. The expanded version increases parameters to 2.036M and GFLOPs to 9.4 but drops AP75 to 30.1, indicating that simply increasing channel capacity introduces redundant features and weakens localization stability. The residual version maintains the baseline computational cost but decreases AP and AP75, suggesting that modifying only the information propagation path is insufficient. Moreover, the combined residual and expanded version incurs higher computational overhead without surpassing the overall AP and APs of the standard LSBlockS. Therefore, the standard LSBlockS design achieves the optimal balance among accuracy, efficiency, and small-object representation.

To analyze the effect of CMP at different network stages, this study configured three intervention strategies—late, mid, and early—on the same lightweight benchmark network. The CMP module structure remained consistent across all variants, with only the embedding position altered. Specifically, late intervention denotes embedding CMP at the high-level semantic stage, mid intervention denotes embedding CMP at the intermediate feature stage, and early intervention denotes embedding CMP at the shallow high-resolution feature stage. The experimental results are shown in Table 6.

The quantitative results indicate that the timing of CMP intervention has a clear impact on detection performance. CMP-EARLY achieves the best overall results, with AP, AP50, AP75, APs, APm, and F1 reaching 42.8, 95.1, 26.0, 41.7, 51.6, and 94.5, respectively. Compared with CMP-LATE, CMP-EARLY improves AP by 1.8 percentage points, APs by 1.6 percentage points, APm by 1.8 percentage points, and F1 by 2.0 percentage points. Meanwhile, the parameter count increases only slightly from 2.92M to 2.95M, and the computational cost increases from 9.7 to 9.9 GFLOPs, indicating that early CMP intervention brings consistent performance gains with only minimal additional overhead. CMP-MID performs between CMP-LATE and CMP-EARLY, suggesting that earlier cross-modal calibration is more effective for enhancing feature representation.

**Figure 9:**
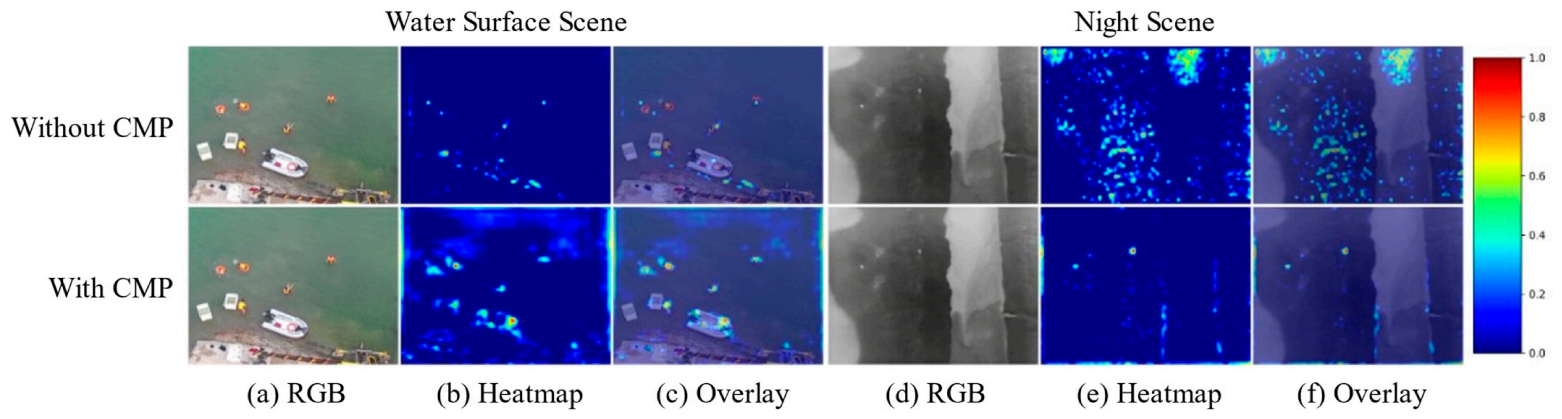
Heatmap visualization of CMP intervention at different feature stages.

Figure 9 illustrates the feature responses arranged by different scenarios and model configurations. The figure is divided into a water surface scene on the left and a night scene on the right. For both scenes, the upper row displays the feature responses without the CMP module at the P3 level, which corresponds to mid-stage or late-stage fusion, while the lower row shows the responses after introducing the CMP module at the P3 level. Within each scene, the columns sequentially show the original RGB image (a) and (d), the isolated attention heatmap (b) and (e), and the heatmap superimposed onto the RGB image (c) and (f). A color bar is provided on the far right to indicate the normalized activation intensity from low to high. When CMP is not introduced at the P3 level, the feature responses are relatively scattered and noticeable background activations remain in cluttered regions. This indicates that delayed cross-modal fusion is insufficient to fully suppress modality-specific noise and spatial response inconsistency accumulated during downsampling. In contrast, after introducing CMP at the P3 level, the target regions exhibit more compact and prominent activations, while redundant background responses are clearly reduced. This demonstrates that early cross-modal prompting at the high-resolution feature stage can better exploit infrared thermal saliency and visible structural details before fine spatial information is lost. Consequently, CMP-EARLY provides cleaner and more discriminative multi-modal features for subsequent pyramid aggregation and detection.

In summary, because early intervention achieves superior performance with minimal additional overhead, it is adopted as the default configuration in the final AFC-Det model. This confirms that introducing cross-modal prompting at the shallow high-resolution stage effectively suppresses early noise and preserves complementary RGB-IR cues for small-target detection.

**Figure 10:**
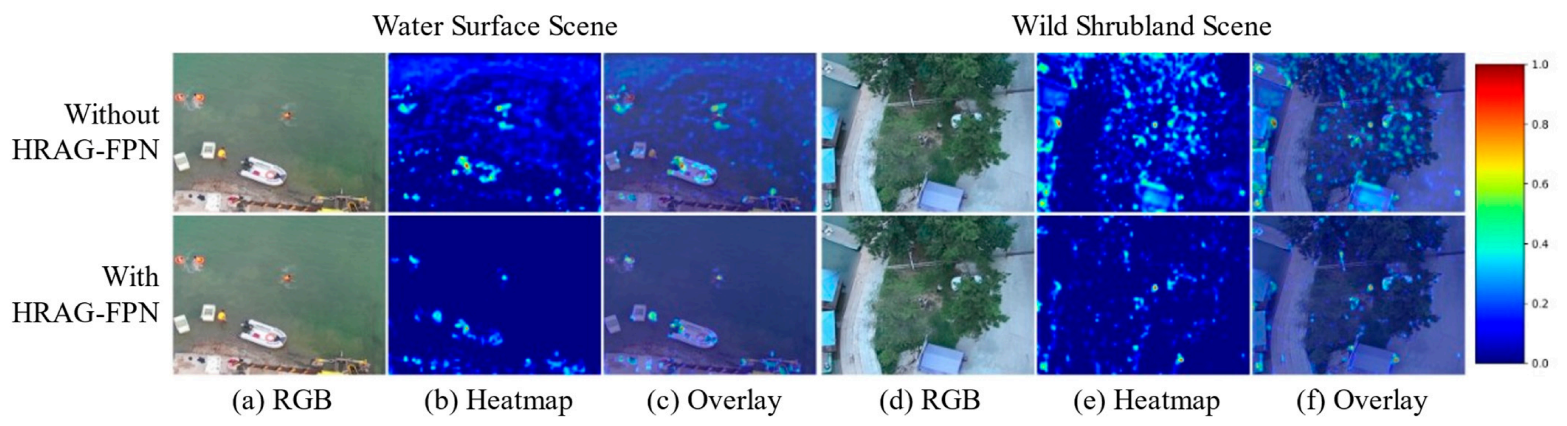
Heatmap visualization of feature responses with and without HRAG-FPN.

To further validate the role of HRAG-FPN in multi-scale feature aggregation, this study performs a heatmap visualization comparison between the model without HRAG-FPN and the complete AFC-Det. Following the same visual arrangement as Figure 9, Figure 10 evaluates the impact of the HRAG-FPN module, displaying the feature responses before and after its integration. The left side depicts a water surface scene, while the right side shows a wild shrubland scene. Without HRAG-FPN, the model’s target responses are relatively dispersed, and small-scale, low-SNR target regions exhibit weak activation. This demonstrates that relying solely on local feature calibration is insufficient for fully capturing tiny targets in complex scenarios.

In contrast, after introducing HRAG-FPN, the heatmap responses in target regions become more concentrated, redundant activations in background regions are reduced, and both small and medium-scale targets exhibit clearer response distributions. This demonstrates that HRAG-FPN can strengthen the interaction between shallow spatial details and deep semantic information through high-resolution anchored aggregation and bottom-up detail feedback, thereby improving small-object feature representation and boundary localization capability.

The visualization results align with the quantitative experiments. After introducing HRAG-FPN, the model achieves clear improvements in AP, AP75, and APs, confirming that this module not only enhances multi-scale target responses but also helps reduce missed detections under complex backgrounds. Therefore, HRAG-FPN contributes positively to improving the completeness of small-object detection by AFC-Det in UAV-SAR scenarios.

#### 3.2.3. Generalization Capability on the M3FD Dataset

To ensure a rigorous and fair evaluation of cross-dataset generalization, all compared networks alongside the proposed AFC-Det were initialized with COCO pre-trained weights and independently trained on the M3FD dataset. The training setup on M3FD strictly adopted identical hyperparameter configurations, optimization strategies, learning rate schedules, and data augmentation recipes as those used in the main VTSaR experiments. This standardized protocol guarantees that the cross-dataset evaluation metrics objectively reflect the architectural generalization and multi-scenario adaptability of each framework across distinct multi-modal data distributions under uniform optimization conditions.

The generalization experiment results on the M3FD dataset are shown in Table 7. AFC-Det achieves the best overall performance in cross-dataset testing, with AP, AP50, and AP75 reaching 54.6%, 86.1%, and 56.8%, respectively, all outperforming the competing methods. Compared with YOLOv11-RGBT, AFC-Det improves AP by 3.0%, AP50 by 3.0%, and AP75 by 2.1%, indicating that the proposed method is not only effective on the original SAR dataset but also demonstrates strong generalization capability on more complex multi-scenario RGB–IR datasets.

In terms of model complexity, AFC-Det contains only 1.7M parameters and 8.0 GFLOPs, significantly lower than YOLOv11-RGBT, DEYOLO, DAMSDet, QFDet, and COXNet. Although DAMSDet, QFDet, and COXNet have larger model sizes and computational costs, their detection performance is noticeably lower than that of AFC-Det, demonstrating that simply increasing network capacity does not effectively enhance cross-scenario multi-modal detection performance. This validates the superior efficiency of our cross-modal feature modeling and lightweight structural design.

Overall, these M3FD results highlight the strong multi-scenario adaptability of AFC-Det, confirming its potential for robust deployment on resource-constrained UAV perception platforms without relying on a single dataset distribution.

### 3.3. Performance Assessment of UAV-Assisted Localization

While the detection frontend AFC-Det was rigorously benchmarked on the large-scale VTSaR dataset to ensure robust statistical evaluation, such public datasets typically lack the synchronized, high-precision UAV telemetry and localized DEM data required for precise 3D geometric localization. Consequently, to validate the overall pipeline, we conducted sequential system evaluations on our custom real-world flight sequences, such as Vis_let1 and polje, which represent typical SAR scenarios.

In these evaluations, raw UAV imagery was first processed by pre-trained AFC-Det to generate bounding box predictions that inherently carry real-world detection noise and sub-pixel jitter. The predictions were then fed directly into the GCR-HRCO backend to compute final 3D geographic coordinates. The robust localization accuracy of these coupled experiments demonstrates the practical viability of our full perception-to-localization pipeline in actual field deployments.

In the localization experiments, the DEM provided by the SAR-DAG_raycast dataset serves as the terrain prior for ray-constrained geographic positioning. This setting assesses the proposed method under a practical UAV-SAR deployment condition, where target localization depends on available terrain information rather than specially surveyed high-resolution elevation data. Since the proposed GCR-HRCO estimates target positions through multi-ray geometric consistency and uses DEM intersections mainly for terrain-constrained height stabilization, the experiment centers on whether the method can yield stable geographic outputs from AFC-Det detections and UAV metadata.

Specifically, the target image points were obtained directly from the bounding boxes predicted by AFC-Det. Each predicted target was converted into a camera ray using the corresponding UAV GPS position, camera parameters, flight attitude, and gimbal metadata. The generated rays were then intersected with DEM terrain to estimate target geographic coordinates. The ablation study included three independent modules, namely Global Context Ray Aggregation (G), Ray Consistency Optimization (R), and Huber Robust Loss (H), resulting in eight combinations. DEM-based terrain-constrained output was used as a fixed post-processing step for all settings. For ray–terrain intersection, the maximum raycasting range was set to 5000 m, the coarse search step to 1.0 m, and the bisection iteration number to 30. In multi-ray optimization, at least three rays were required, the Iteratively Reweighted Least Squares (IRLS) iteration number was set to 8, the Huber threshold was set to 3.0 m, and the regularization coefficient was set to $1\times 10^{-6}$.

#### 3.3.1. Quantitative Analysis of Localization Accuracy

To evaluate the contributions of different localization modules, ablation experiments were conducted using AFC-Det predicted bounding boxes as the visual input. As shown in Table 8, the baseline setting without global context aggregation, ray consistency optimization, or Huber robust loss yielded a mean error of 6.85 m and a root mean square error, denoted as RMSE, of 8.16 m. This indicates that simple short-sequence ray averaging is highly sensitive to detection noise and unstable viewpoints. While introducing individual modules improved accuracy to varying degrees, the combination of global context aggregation and ray consistency optimization produced the most significant dual-module improvement, drastically reducing both mean error and RMSE.

Integrating all three components achieved the best overall performance. Compared with the baseline, the mean error and RMSE were reduced by 51.7% and 46.9% respectively, alongside significant drops across all percentile error metrics including P90 and P95. These results confirm that global context aggregation expands the observation range, ray consistency optimization strengthens multi-ray geometric constraints, and the Huber robust loss suppresses inconsistent rays. Their synergistic combination substantially improves the accuracy and stability of UAV-SAR target localization under visual detection perturbations.

While the proposed GCR-HRCO localization backend demonstrates significant accuracy improvements, we acknowledge that the current evaluation is based on a relatively limited number of flight sequences and distinct target locations. This limitation stems from the immense difficulty of acquiring large-scale public datasets that simultaneously provide synchronized UAV telemetry, camera metadata, localized terrain models, and highly accurate geographic ground truth.

However, it is crucial to emphasize that the generalization capability of the proposed localization framework is fundamentally robust. Unlike data-driven deep learning models that are prone to overfitting to specific visual distributions, our localization backend is strictly grounded in deterministic multi-view geometry and robust mathematical optimization. Consequently, its adaptability to more diverse environments does not depend on the visual appearance or texture of the rescue scene. Instead, the system’s generalization is primarily governed by flight kinematics and geometric constraints. We expect the proposed approach to generalize seamlessly to highly diverse search and rescue scenarios provided that the UAV executes adequate lateral movement to establish multi-ray parallax and a baseline DEM is available. Future work will focus on collecting more extensive flight data across varied topographies to systematically quantify the geometric boundaries of this system.

#### 3.3.2. Qualitative Assessment and Visualizations

**Figure 11:**
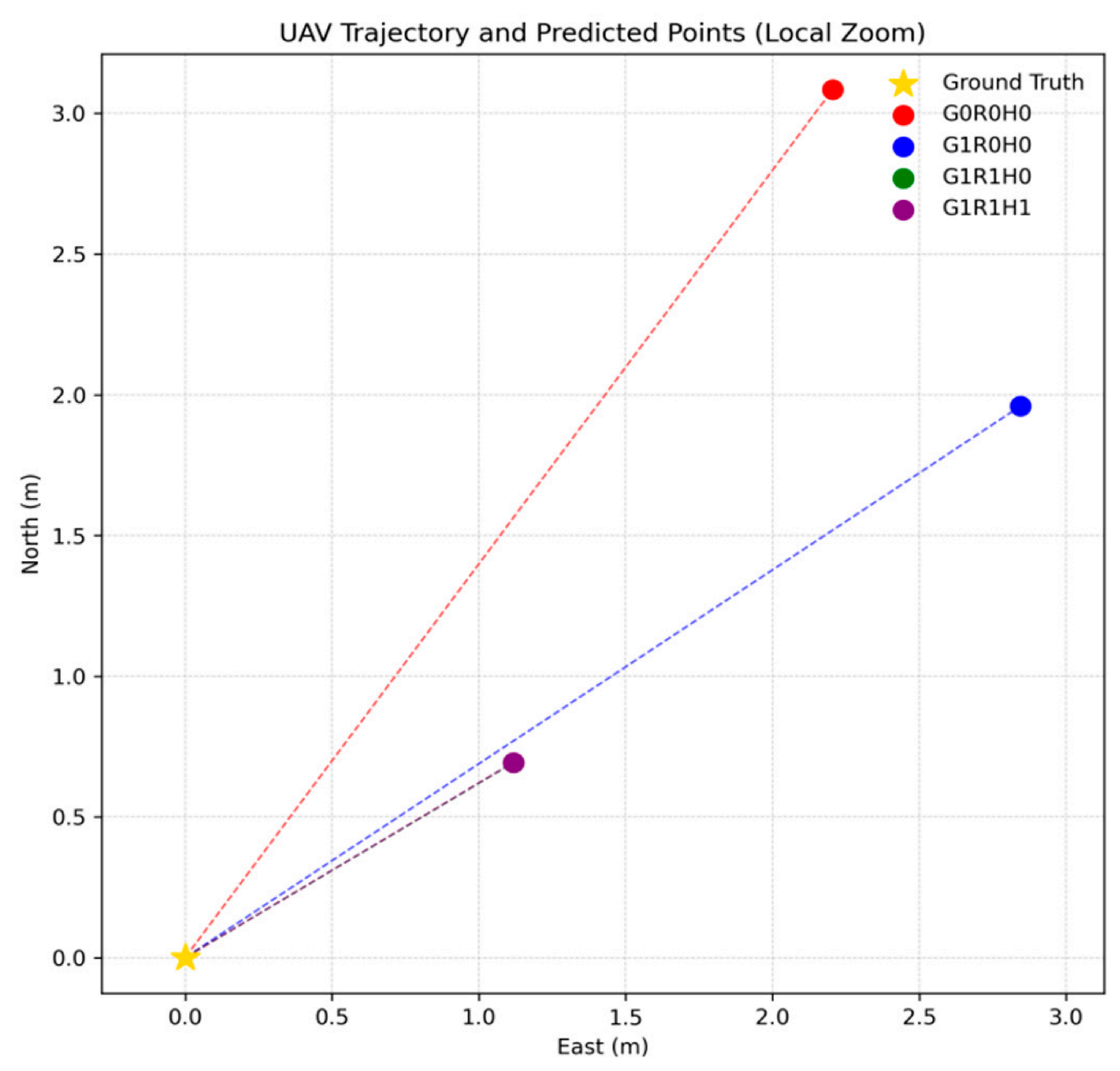
Geographic visualization of UAV-assisted localization under different GCR-HRCO module configurations. The zoomed-in local plot displays a spatial region with an axis range of 0–3 m relative to the ground-truth target. The green point G1R1H0 overlaps with G1R1H1 and is visually obscured.

To further illustrate the impact of the proposed modules on UAV-assisted localization, we provide qualitative visualizations of a representative flight sequence. As shown in Figure 11, the ground-truth target is indicated by a yellow star, and predicted localization points from different module combinations are shown in distinct colors.

The figure is locally zoomed to illustrate the spatial differences among representative estimates. The baseline G0R0H0 estimate is located farthest from the ground truth, reflecting the limitations of short-sequence ray averaging under detection perturbations. Introducing global context aggregation noticeably shifts the G1R0H0 estimate closer to the target, while adding multi-ray geometric consistency further pulls the G1R1H0 estimate toward the ground-truth position. Although the incremental effect of the Huber robust loss in the complete G1R1H1 configuration is not visually distinguishable in this specific example, the overall error statistics in Table 8 confirm its absolute quantitative superiority. These results demonstrate how the proposed components jointly improve the overall accuracy and robustness of UAV-SAR target localization.

**Figure 12:**
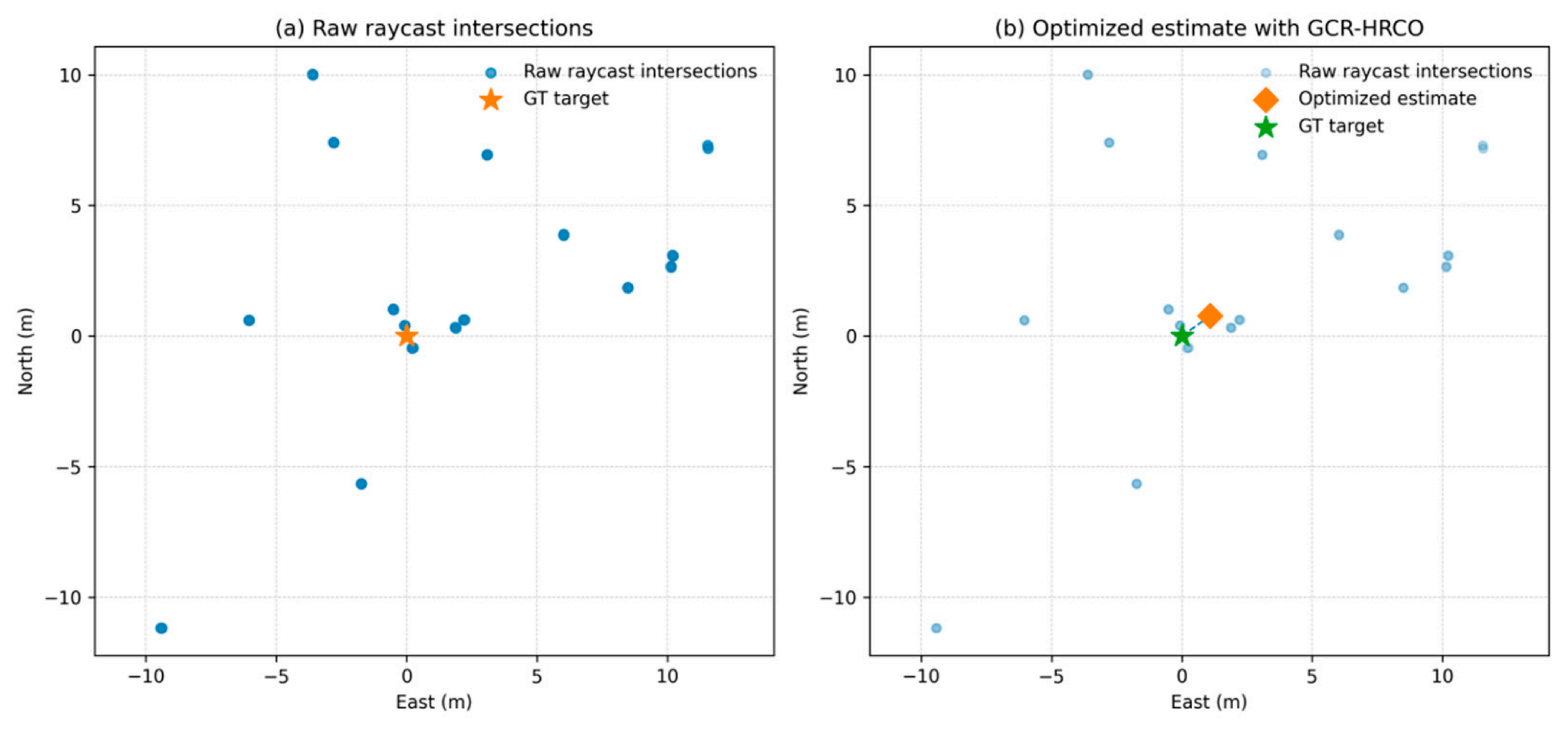
Ray-consistency analysis before and after GCR-HRCO optimization.

To further evaluate the effectiveness of DEM constraints and multi-ray geometric consistency, Figure 12 visualizes the spatial distribution of raycast intersections before and after optimization. The left panel shows the raw raycast intersections generated directly from AFC-Det bounding boxes. These intersections are severely scattered around the ground-truth target due to detection deviations, viewpoint variations, and the instability of single-frame ray-terrain intersections. Conversely, the right panel presents the optimized localization result obtained by applying the complete GCR-HRCO pipeline. After optimization, the predicted location is successfully pulled toward the geometrically consistent region formed by multiple observation rays, aligning much more closely with the ground-truth target. Compared with the raw raycast intersections, this optimized result demonstrates higher stability and precision, confirming that the proposed pipeline effectively refines UAV-assisted target localization in complex SAR scenarios.

**Figure 13:**
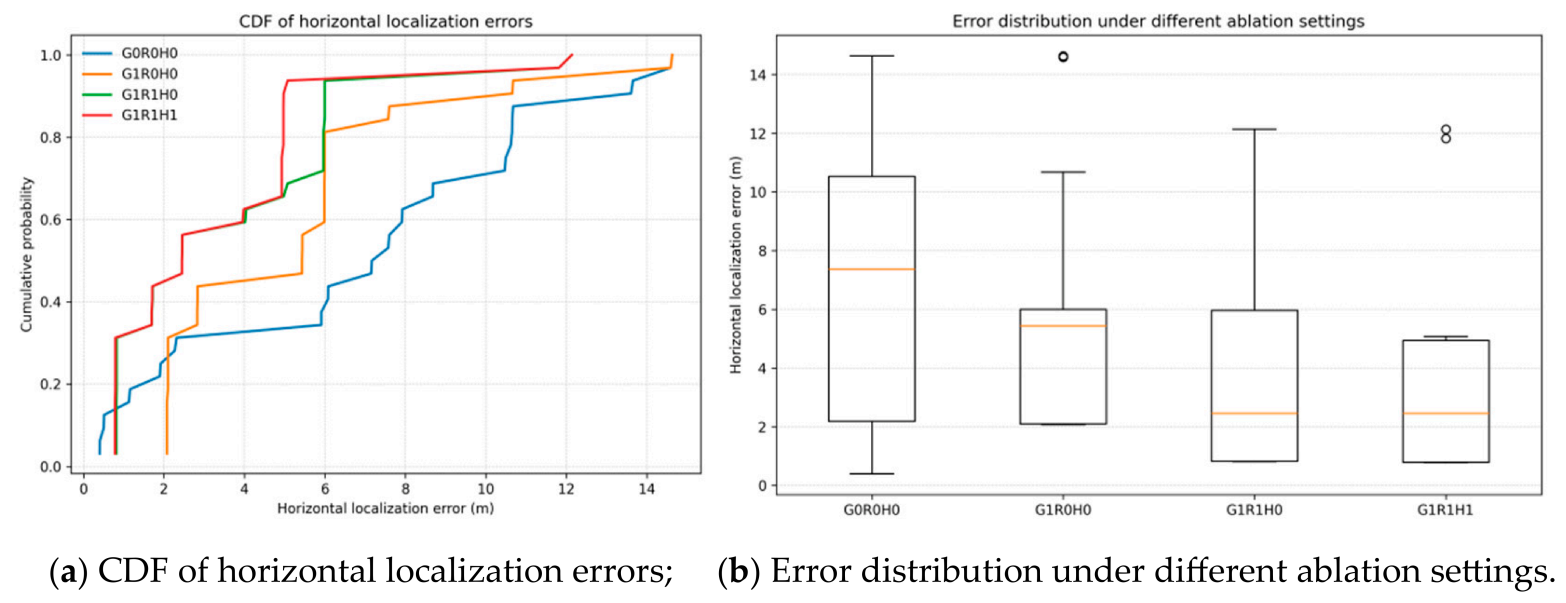
Horizontal localization error distributions under different GCR-HRCO ablation settings.

To quantitatively assess localization accuracy and robustness across different module configurations, the horizontal localization error distributions are analyzed using cumulative distribution functions (CDFs) and boxplots, as shown in Figure 13. The CDF curves in Figure 13a show that the baseline configuration (G0R0H0) converges most slowly, with a substantial proportion of samples exhibiting errors greater than 6 m. Introducing global context aggregation (G1R0H0) shifts the CDF leftward, indicating an overall reduction in localization error. With the addition of multi-ray geometric consistency, G1R1H0 further reduces the median error, and most predictions fall within 5 m. The CDF of the complete GCR-HRCO configuration (G1R1H1) largely overlaps that of G1R1H0 in the lower-error range but rises more rapidly at higher cumulative probabilities, reflecting improved upper-tail performance.

Figure 13b complements this analysis by visualizing the overall error spread under each setting. The baseline exhibits the highest median error and the broadest distribution. While global context aggregation and geometric consistency drastically reduce the median error, the complete G1R1H1 configuration uniquely achieves a lower upper whisker and tighter high-percentile bounds despite a few remaining outliers. Together with the quantitative metrics reported previously, these error distribution analyses confirm that combining all three components effectively improves average localization accuracy while enhancing robustness against inconsistent observations in complex rescue scenarios.

Table 9 compares GCR-HRCO with six conventional localization baselines under identical experimental conditions, including AFC-Det direct detection outputs, UAV metadata, DEM data, and temporal sequence partition. Each method generated one localization estimate for each of the 16 temporal sequences, and all methods achieved a valid localization rate of 100%. GCR-HRCO consistently outperformed all competitors. Compared with RANSAC multi-ray fitting, the strongest conventional baseline, GCR-HRCO reduced the mean error and RMSE by 39.1% and 33.1% respectively, achieving the lowest overall errors across all metrics. The lower P90 and P95 errors further demonstrate its improved robustness against large localization deviations.

To elucidate the physical boundaries of our vision-based localization framework, we conducted an in-depth analysis of a representative failure case within Sequence 0 from the Vis_let1 flight. Quantitative metrics from this sequence reveal a severe operational condition where the mean UAV-to-target raycast distance reached 80.31 m. Notably, the internal Ray Consistency Optimization module performed exceptionally well, successfully aggregating the camera rays and yielding a multi-ray perpendicular residual of merely 1.73 m. This low residual indicates that the extracted spatial rays intersected tightly and consistently in 3D space.

Despite this optimal internal convergence, the final absolute 3D positioning error surged to 11.91 m. A detailed decomposition of this error uncovered a stark spatial contrast: the vertical elevation error along the *Z*-axis was relatively constrained at 1.52 m, whereas the horizontal planar error on the XY-plane dominated at 11.81 m.

This disproportionate horizontal drift perfectly illustrates the classic geometric lever-arm effect inherent in purely vision-based systems. At an extreme projection range exceeding 80 m, even sub-pixel fluctuations in the bounding box predicted by the detection frontend translate into minute angular deviations of the optical rays. Projected over this massive lever arm onto the terrain, these micro-angular errors are geometrically magnified into massive horizontal translations. This data-driven case explicitly demonstrates that while our multi-view optimization ensures tight ray convergence, the absolute geographical accuracy remains fundamentally bounded by optical resolution limits and the geometric magnification at high altitudes.

Evaluating practical UAV deployment requires analyzing the end-to-end runtime across the complete perception-to-localization pipeline rather than evaluating the detector in isolation. While the frontend detector dominates the processing time by requiring roughly 9.3 ms per frame on a desktop NVIDIA RTX 3090 GPU, the GCR-HRCO localization backend is remarkably lightweight. Because this backend processes sparse geometric coordinates rather than dense visual tensors, its computational footprint consumes less than 1 ms on a desktop platform. To explicitly demonstrate its suitability for real-time edge processing, the localization backend was benchmarked on a highly resource-constrained 168 MHz STM32F407 microcontroller without hardware acceleration, where converging the complete spatial sequence required merely 5.3 ms. This millisecond-level efficiency guarantees that multi-view geometric optimization will not become a bottleneck even on low-power bare-metal hardware. Although the vision frontend evaluation currently relies on a desktop GPU, the proven efficiency of the localization backend confirms the architectural viability of the entire pipeline, establishing a concrete foundation for full onboard integration on edge platforms such as Jetson-class devices.

Regarding practical deployment factors, the real-world operational behavior of the system extends beyond pure computational speed. Under an online streaming mechanism, each newly captured image dynamically updates the observation queue. Consequently, a clear system-level distinction must be made between computational latency and physical kinematic accumulation time. The actual geographic output cadence is strictly governed by the physical flight time needed for the UAV to travel and establish sufficient spatial baselines. This physical data acquisition rate, rather than the algorithmic execution speed, dictates the true operational rhythm during practical edge deployment.

One limitation of this study lies in the absence of a unified UAV-assisted SAR benchmark that simultaneously provides aligned RGB–IR imagery, target detection annotations, UAV pose information, camera and gimbal parameters, DEM data, and ground-truth geographic target locations. To address this dataset gap, the proposed framework was evaluated in a staged manner. The multispectral detection model was trained and tested on visual RGB–IR datasets, while the geographic localization module was evaluated on the SAR-DAG_raycast dataset, which provides UAV metadata and geographic reference information. For AFC-Det inference on SAR-DAG_raycast, each RGB frame was paired with a spatially aligned infrared-domain representation obtained using DR-AVIT, forming the required RGB–IR input. This evaluation strategy effectively bridges the perception and localization components under existing data constraints. Nevertheless, the reported results should be regarded as a staged perception-to-localization evaluation rather than a unified end-to-end multispectral UAV benchmark.

Another limitation is that only coarse-resolution DEM data were available for the SAR-DAG_raycast localization experiments. Although this setting represents practical UAV-SAR conditions, where high-resolution DEMs are often difficult to obtain in remote or disaster-affected areas, it prevents a systematic sensitivity analysis comparing coarse and high-precision terrain models. Future work will focus on constructing or collecting a unified RGB–IR UAV-SAR dataset with complete detection, pose, terrain, and geographic annotations, and will further evaluate the sensitivity of the proposed GCR-HRCO framework to DEM resolution and terrain data quality.

Additionally, it should be noted that the current performance evaluation represents an offline software validation rather than an onboard deployment. The reported inference speed and system latency were measured on a desktop-class NVIDIA RTX 3090 GPU, which possesses computing power and memory bandwidth vastly exceeding those of typical resource-constrained UAV payloads. Although the proposed framework demonstrates highly promising lightweight theoretical metrics, including a reduced parameter count and low GFLOPs, these results serve as a preliminary validation of our architectural efficiency. Consequently, translating these theoretical advantages into practical edge performance requires dedicated hardware acceleration, and conducting rigorous onboard real-time validation on representative embedded hardware remains a primary objective for our future work.

## 4. Conclusions

This paper proposes a unified perception-to-localization framework for UAV-assisted SAR. The proposed framework couples lightweight RGB-IR small-target detection with DEM-constrained ray consistency localization, enabling the robust and efficient conversion of detected targets into geographic coordinates. In the perception stage, AFC-Det improves the reliability of small-target visual observations by leveraging complementary RGB structural cues and infrared thermal saliency. In the localization stage, GCR-HRCO enhances target-level geolocation by aggregating flight-level rays and enforcing multi-ray geometric consistency under terrain constraints. Experiments on VTSaR and M3FD validate the accuracy and efficiency of the proposed multispectral detector, while localization results on SAR-DAG_raycast under the constructed RGB-IR input setting show that the localization pipeline reduces the mean horizontal error from 6.85 m to 3.31 m and the RMSE from 8.16 m to 4.33 m. These results confirm that this method offers an effective and practical solution for UAV-assisted SAR perception and geographic localization in complex outdoor environments.

Future work will extend the proposed framework in several directions. First, a unified multispectral UAV-SAR dataset will be constructed to support end-to-end evaluation, including aligned RGB-IR imagery, detection annotations, UAV pose information, camera and gimbal parameters, DEM data, and ground-truth geographic target locations. Second, DEMs with different spatial resolutions and accuracy levels will be introduced to systematically evaluate the influence of terrain data quality on GCR-HRCO. Third, more challenging SAR conditions, such as moving targets, steep terrain, dense canopy, urban occlusions, and UAV pose or gimbal uncertainties, will be considered to further improve the robustness and practical applicability of target-level geographic localization.

## Figures and Tables

**Table 1:** Parameter setting.

Parameter	Value
Image size	640 × 640
Epochs	300
Batch size	32
Optimizer	SGD
Initial learning rate	0.01
Momentum	0.937
Weight decay	0.0005
Mosaic	True
AMP	False
Seed	42
Early Stop	20

**Table 2:** Statistics of the SAR-DAG_raycast evaluation subset and DEM configuration.

Category	Statistic	Value
Data scale	Number of flights	7
Data scale	Number of distinct ground-truth target locations	6
Data scale	Number of temporal sequences	16
Data scale	Number of evaluated frames	154
Ray validity	Number of valid DEM-intersecting rays	154
Ray validity	Valid-ray rate	100.0%
Flight geometry	UAV altitude above ground level	10.23–41.37 m
Flight geometry	Horizontal UAV-to-target distance	3.77–112.36 m
Flight geometry	Slant UAV-to-target distance	17.66–118.29 m
Terrain data	DEM tile	N45E016.hgt
Terrain data	DEM raster size	3601 × 3601
Terrain data	DEM angular sampling interval	1 arc-second
Coordinate representation	Input geographic coordinates	GPS latitude and longitude
Coordinate representation	Metric optimization frame	Local east–north–up (ENU) frame

**Table 3:** Comparison with state-of-the-art on VTSaR dataset.

Model	AP	AP50	AP75	APs	AR@100	Param	GFLOPs	FPS
AFC-Det	45.$4_{-0.2}^{+0.8}$	95.$5_{-0.3}^{+0.9}$	33.$7_{-0.2}^{+0.3}$	44.$7_{-0.1}^{+0.6}$	53.$5_{-0.3}^{+0.6}$	1.7	8.0	107.2
YOLOv11-RGBT [38]	45.$1_{-0.9}^{+0.1}$	95.$8_{-0.7}^{+0.3}$	31.$4_{-0.6}^{+0.7}$	44.$2_{-0.4}^{+0.1}$	53.$3_{-0.6}^{+0.2}$	5.0	11.0	56.7
DAMSDet [39]	43.$9_{-0.5}^{+0.4}$	94.$8_{-0.6}^{+0.4}$	29.$4_{-0.3}^{+0.3}$	43.$3_{-0.6}^{+0.4}$	57.$1_{-0.8}^{+0.3}$	79.1	134.7	21.4
DEYOLO [40]	43.$5_{-0.3}^{+0.6}$	93.$9_{-0.3}^{+0.5}$	29.$5_{-0.7}^{+0.1}$	42.$5_{-0.5}^{+0.2}$	52.$7_{-0.3}^{+0.5}$	6.0	16.8	59.1
QFDet [20]	42.$2_{-0.7}^{+0.3}$	93.$1_{-0.5}^{+0.6}$	28.$2_{-0.4}^{+0.6}$	40.$9_{-0.6}^{+0.3}$	50.$8_{-0.6}^{+0.2}$	60.2	203.6	23.8
COXNet [41]	41.$8_{-0.9}^{+0.1}$	93.$1_{-0.1}^{+0.5}$	26.$8_{-0.2}^{+0.6}$	40.$6_{-0.2}^{+0.7}$	49.$4_{-0.4}^{+0.3}$	71.1	128.1	24.7

**Table 4:** Ablation experiment on VTSaR dataset.

Model Variant	Params/M	GFLOPs	AP	AP50	AP75	APs	APm	F1
Baseline	2.89	9.4	42.2	95.0	26.5	41.1	49.4	94.3
+CMP	2.95	9.9	42.8	95.1	26.0	41.7	51.6	94.5
+HRAG-FPN	3.13	9.7	44.5	95.7	30.7	43.8	50.6	95.1
+Block	1.35	7.1	41.5	93.8	24.0	40.6	47.9	93.7
+C + B	1.41	7.6	42.7	94.2	26.4	42.0	49.5	94.1
+C + H	3.55	11.4	44.9	95.5	30.9	44.1	50.8	95.2
+B + H	1.59	7.5	44.9	95.5	32.2	44.2	50.6	95.0
AFC-Det	1.65	8.0	45.4	95.5	33.7	44.7	51.6	95.4

**Table 5:** Ablation study of modality-specific encoding blocks and LSBlockS variants on the VTSaR dataset.

Group	RGB Branch	Thermal Branch	Variant	Params (M)	GFLOPs	AP	AP50	AP75	APs	APm	AR@100
A	BasicBlock	BasicBlock	BasicBlock Variant	3.551	11.4	44.9	95.5	30.9	44.1	50.8	52.7
A	StarBlock	BasicBlock	RGB enhanced	2.579	9.1	44.8	95.7	31.3	44.0	52.0	53.2
A	BasicBlock	LSBlockS	Thermal enhanced	2.263	9.1	44.2	95.5	28.9	43.3	50.6	52.1
A	StarBlock	LSBlockS	Standard	1.650	8.0	45.4	95.5	33.7	44.7	51.6	53.5
B	StarBlock	LSBlockS	Expanded	2.036	9.4	45.2	95.7	30.1	44.6	50.7	53.2
B	StarBlock	LSBlockS	Residual	1.650	8.0	44.8	95.4	30.6	44.0	50.9	52.8
B	StarBlock	LSBlockS	Residual + Expanded	2.036	9.4	45.2	95.3	32.1	44.3	52.1	53.4

**Table 6:** Effect of CMP intervention timing on VTSaR dataset.

Model Variant	Params/M	GFLOPs	AP	AP50	AP75	APs	APm	F1
CMP-LATE	2.92	9.7	41.0	93.7	24.6	40.1	49.8	92.5
CMP-MID	2.93	9.8	42.5	94.2	25.4	41.5	51.0	93.9
CMP-EARLY	2.95	9.9	42.8	95.1	26.0	41.7	51.6	94.5

**Table 7:** Cross-dataset generalization comparison on the M3FD dataset.

Model	AP	AP50	AP75	Param	GFLOPs
AFC-Det	54.6	86.1	56.8	1.7	8.0
YOLOv11-RGBT [38]	51.6	83.1	54.7	5.0	11.0
DAMSDet [39]	48.4	75.8	49.3	79.1	134.7
DEYOLO [40]	49.4	72.3	54.8	6.0	16.8
QFDet [20]	45.4	72.5	47.0	60.2	203.6
COXNet [41]		72.9	42.8	71.1	128.1

**Table 8:** Ablation study of GCR-HRCO modules for UAV-assisted geographic localization.

Setting	G	R	H	Mean Error (m)	Median Error (m)	RMSE (m)	P90 Error (m)	P95 Error (m)
G0R0H0				6.849	7.362	8.160	12.166	13.890
G1R0H0	√			5.239	5.430	6.250	9.126	11.656
G0R1H0		√		5.522	4.270	6.866	11.770	12.919
G0R0H1			√	6.053	6.367	7.212	9.781	10.923
G1R1H0	√	√		3.577	2.447	4.649	5.997	7.452
G1R0H1	√		√	4.452	4.804	5.633	8.136	10.923
G0R1H1		√	√	5.366	4.270	6.540	11.440	12.475
G1R1H1	√	√	√	3.305	2.447	4.329	4.970	6.684

**Table 9:** Comparison with conventional localization baselines on the SAR-DAG_raycast dataset.

Method	Mean Error (m)	Median Error (m)	RMSE (m)	P90 Error (m)	P95 Error (m)
Single-frame DEM raycasting	6.696	6.942	7.918	10.877	12.053
Short-sequence intersection mean	6.849	7.362	8.160	12.166	13.890
Short-sequence intersection median	6.812	7.351	8.081	11.798	12.910
Ordinary multi-ray least squares	6.025	4.604	7.711	13.196	14.904
RANSAC multi-ray fitting	5.426	4.604	6.467	11.513	12.038
Huber M-estimator	6.003	4.604	7.651	13.213	14.765
GCR-HRCO (proposed)	3.305	2.447	4.329	4.970	6.684

## Data Availability

The data presented in this study are available on demand from the corresponding author at (zcs@sdust.edu.cn).
